# Comparative Analysis of Endothelial Cell Culture Models Under Altered Mechanical Conditions and Glucose Variations

**DOI:** 10.3390/ijms27146233

**Published:** 2026-07-13

**Authors:** Augusta Volkevičiūtė, Jayashree Sahana, Estéfano Pinilla, Daniela Melnik, Luis Fernando González-Torres, Markus Wehland, Edgaras Stankevicius, Daniela Grimm, Ulf Simonsen

**Affiliations:** 1 Preclinical Research Laboratory for Medicinal Products, Institute of Cardiology, Lithuanian University of Health Sciences, 50162 Kaunas, Lithuania; edgaras.stankevicius@lsmu.lt (E.S.); us@biomed.au.dk (U.S.); 2 Department of Biomedicine, Pulmonary and Cardiovascular Pharmacology, Aarhus University, 8000 Aarhus, Denmark; jaysaha@biomed.au.dk (J.S.); estefanopinilla@biomed.au.dk (E.P.); dgg@biomed.au.dk (D.G.); 3 Hematopoiesis and Leukocyte Biology Lab, Baker Heart and Diabetes Institute, Melbourne 3004, Australia; 4 Department of Microgravity and Translational Regenerative Medicine, Otto von Guericke University, 39106 Magdeburg, Germany; daniela.grimm@med.ovgu.de (D.M.); luis.gonzaleztorres@med.ovgu.de (L.F.G.-T.); markus.wehland@med.ovgu.de (M.W.); 5 Research Group “Magdeburger Arbeitsgemeinschaft für Forschung unter Raumfahrt- und Schwerelosigkeitsbedingungen” (MARS), Otto von Guericke University, 39106 Magdeburg, Germany

**Keywords:** endothelial cells, hyperglycemia, 3D clinostat, multicellular spheroids, angiogenesis, cell adhesion molecules

## Abstract

Endothelial dysfunction is a defining feature of diabetic vascular disease and is characterized by impaired nitric oxide signaling, inflammatory activation, altered mechanotransduction, and disturbed angiogenic responses. The present study investigated whether hyperglycemia modulates endothelial phenotype in a model-dependent manner under distinct structural and mechanical culture conditions. EA.hy926 endothelial cells were cultured for 7 days under normoglycemic (5.5 mM) or high-glucose (25 mM) conditions as static monolayers (G-force = 1 *g*), adherent clinorotated cells, multicellular spheroids (MCSs) generated during clinorotation, and Matrigel-derived endothelial structures. Gene expression was assessed by qPCR using marker panels related to nitric oxide signaling, *PI3K CA-AKT-mTOR* signaling, inflammatory adhesion, angiogenesis, and structural adhesion, whereas protein abundance and spatial distribution of eNOS, AKT1, VCAM1, vinculin, and VEGFA together with CDH5/VE-cadherin were analyzed by Western blotting and confocal microscopy. Clinorotation generated both adherent endothelial cells and MCSs. High glucose reduced eNOS-related expression in most models, with the strongest decreases in adherent clinorotated cells and MCSs, whereas Matrigel cultures showed a divergent transcriptional response. PI3K CA expression was markedly suppressed by high glucose in clinostat-derived populations, while *mTOR* was differentially regulated in spheroids and Matrigel cultures. VCAM1 expression was most prominent in MCSs, whereas *ICAM1* was highest in Matrigel cultures. VEGF-related signaling differed substantially among models, and Matrigel cultures showed the strongest VEGFA-associated protein signal and the clearest angiogenic organization under normoglycemic conditions, which became less distinct under high glucose. Vinculin protein abundance was highest in MCSs and Matrigel cultures, reflecting pronounced differences in structural organization. Overall, these findings show that endothelial responses to hyperglycemia are strongly shaped by mechanical and structural context and support the use of complementary in vitro models for studying diabetic endothelial dysfunction.

## 1. Introduction

Endothelial cells (ECs) form the inner lining of blood vessels (intima) and maintain vascular homeostasis by regulating vasodilation, blood flow, immune cell trafficking, and barrier integrity [1,2,3]. In vivo, endothelial cells are constantly exposed to biochemical signals and mechanical forces such as shear stress from blood flow, which shapes their phenotype and function [4,5,6]. Disturbances in these regulatory mechanisms contribute to vascular pathologies, particularly under hyperglycemic conditions, by impairing nitric oxide bioavailability and promoting inflammation through increased oxidative stress and adhesion molecule expression [7,8,9,10,11,12].

Traditional static two-dimensional (2D) monolayer cultures of ECs, although widely used, fail to recapitulate the complex physiological environment of the vasculature [13,14,15]. Cells in 2D adopt a flattened morphology, lack proper cell–cell and cell–matrix interactions, and do not experience shear stress, which is known to upregulate endothelial nitric oxide synthase (*eNOS*) and align cytoskeletal structures [4,5,13,14,15]. More advanced models have been introduced to overcome these limitations, including multicellular spheroids (MCSs), Matrigel-based angiogenesis assays, and mechanical stimulation systems such as clinostat rotation [16,17,18,19,20]. These models capture distinct structural and organizational features of endothelial behavior that are not accessible in simple static monolayer cultures, thereby improving the contextual relevance of in vitro experiments for vascular research [14,18].

MCSs provide a three-dimensional (3D) environment that reproduces cell–cell interactions and nutrient gradients [17,18]. They are widely used to study angiogenesis, particularly through sprouting assays in response to VEGF stimulation, and have been shown to induce distinct transcriptional programs compared with monolayer cultures [14,17,18]. ECs grown on Matrigel rapidly form capillary-like structures and were therefore used here as a model of matrix-guided endothelial organization and early angiogenic patterning, while recognizing that Matrigel represents a biologically complex angiogenic environment rather than a compositionally defined extracellular matrix system [19,20,21]. The 3D clinostat, by continuously altering the orientation of the gravity vector, imposes a mechanically induced change in the culture environment on adherent ECs and enables the formation of 3D spheroids under continuous rotation. These mechanical stimuli alter cytoskeletal organization, focal adhesion distribution, and *eNOS* expression, making clinostat-based cultures particularly useful for investigating mechanotransduction [16,22,23].

Recent evidence highlights the interplay between hyperglycemia and simulated microgravity. Jokšienė et al. (2023) demonstrated that EA.hy926 ECs exposed to both high glucose and clinostat culture exhibited more pronounced changes in gene expression, extracellular matrix remodeling, and oxidative stress than when exposed to either condition alone [24]. The study also showed that microgravity enhanced high-glucose–induced endothelial apoptosis and modulated angiogenesis-related pathways. These findings emphasize the need to study endothelial models under combined metabolic and mechanical challenges to better approximate diabetic vascular dysfunction [22,23,24].

This study was designed to compare how the same endothelial cell type responds to glucose stress when maintained in four distinct structural and mechanical states: static monolayers, adherent clinorotated cultures, multicellular spheroids generated under the same clinostat conditions, and matrix-guided endothelial structures in Matrigel. Whereas previous endothelial clinostat studies have primarily investigated the effects of simulated microgravity or altered mechanical loading in a single culture configuration [16,22,23,24], the present study directly compares multiple endothelial organizational states generated under related experimental conditions. This approach allows us to determine whether endothelial responses to hyperglycemia are primarily driven by altered mechanical loading itself or are further modified by substrate attachment, three-dimensional aggregation, or matrix-guided angiogenic organization [14,17,18,22,23,24]. Consequently, the study provides practical guidance for selecting endothelial culture models according to the specific biological process under investigation. Importantly, the present design was not intended to reproduce physiological laminar shear stress but rather to determine how altered mechanical loading, substrate attachment, three-dimensional aggregation, and matrix-guided organization modify endothelial phenotype under normoglycemic and high-glucose conditions [14,18,25,26]. We therefore focused on gene and protein expressions of key endothelial markers involved in nitric oxide signaling, PI3K, CA-AKT-mTOR signaling, inflammatory activation, angiogenic regulation, and structural adhesion. eNOS regulates nitric oxide production and vascular tone [11]. VEGFA and its receptor, VEGFR2 (also referred to as FLK1/KDR), mediate angiogenic signaling and endothelial cell proliferation [27,28]. AKT1 integrates survival and pro-angiogenic signaling and is functionally linked to endothelial nitric oxide signaling [11,27]. Vinculin was included as a mechanosensitive adhesion-associated protein implicated in cytoskeletal force transmission, although integrin-specific markers would provide more direct information on endothelial matrix attachment [29,30,31]. VCAM1 is an adhesion molecule upregulated during inflammatory activation, particularly under hyperglycemic and disturbed-flow conditions [4,12]. These markers provide a framework for assessing how different endothelial models replicate physiological and pathological phenotypes.

## 2. Results

### 2.1. eNOS-Related Gene and Protein Expression

**Figure 1:**
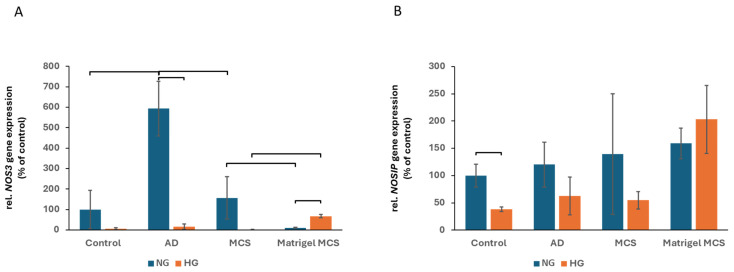
eNOS-related gene expression in EA.hy926 endothelial cells cultured under normoglycemic and high-glucose conditions. Relative mRNA expression of *NOS3* and *NOSIP* was determined in static monolayers (Controls), adherent clinorotated cells (ADs), multicellular spheroids (MCSs), and Matrigel-derived endothelial structures after 7 days of culture under normoglycemic conditions (NG, 5.5 mM) or high glucose (HG, 25 mM). Gene expression was analyzed by qPCR, normalized to 18S rRNA, and expressed relative to the NG Control group using the ΔΔC_T_ method. Bars represent means ± SD. Blue bars indicate NG, and orange bars indicate HG. (**A**) *NOS3*; (**B**) *NOSIP***.** Data are presented as mean ± SD from *n* = 5 independent biological replicates. Overall group differences were analyzed by one-way ANOVA followed by Tukey’s post hoc test. Predefined pairwise comparisons were analyzed using the Mann–Whitney U test. Statistical significance was accepted at *p* < 0.05. Horizontal brackets indicate statistically significant comparisons.

*NOS3* gene expression under NG conditions was significantly elevated in ADs compared to both Control and MCS populations and significantly downregulated in Matrigel vs. MCSs. Under HG conditions, Matrigel MCS cells exhibited significantly higher eNOS expression than MCS cells. While *NOS3* expression was generally lower under HG vs. NG in the Control, AD, and MCS populations, it was significantly increased under HG vs. NG in Matrigel-derived samples. (Figure 1). For *NOSIP*, the only significant difference was observed between HG Controls and NG Controls.

**Figure 2:**
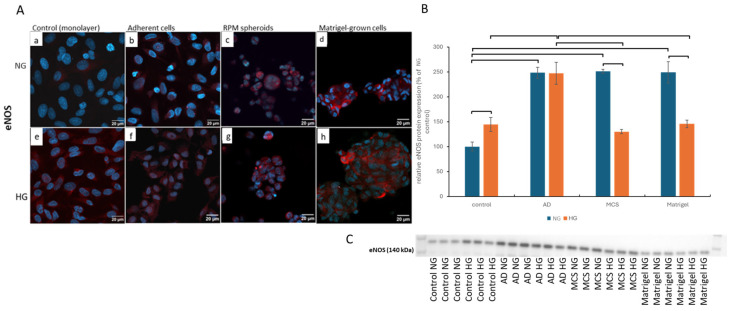
eNOS protein expression and localization in EA.hy926 endothelial cells cultured under normoglycemic and high-glucose conditions. (**A**) Representative confocal images and (**B**) Western blot analysis of eNOS in static monolayers (Controls), adherent clinorotated cells (ADs), multicellular spheroids (MCSs), and Matrigel-derived endothelial structures after 7 days under normoglycemic conditions (NG, 5.5 mM) or high glucose (HG, 25 mM). Confocal images show eNOS immunostaining in red and nuclei counterstained with DAPI in blue. Panels (**a**–**d**) represent NG conditions, and panels (**e**–**h**) represent HG conditions: (**a**,**e**) Control; (**b**,**f**) AD; (**c**,**g**) MCS; (**d**,**h**) Matrigel. Scale bar = 20 µm. The bar graph shows densitometric quantification of eNOS protein expression normalized to the NG Control group. Bars represent means ± SD. (**C**) A representative Western blot is shown below. Unlabeled lanes contain protein markers. Data are presented as mean ± SD from *n* = 5 independent biological replicates. Overall group differences were analyzed by one-way ANOVA followed by Tukey’s post hoc test. Predefined pairwise comparisons were analyzed using the Mann–Whitney U test. Statistical significance was accepted at *p* < 0.05. Horizontal brackets indicate statistically significant comparisons.

Western blot and confocal microscopy confirmed eNOS protein expression in all four endothelial cell models (Figure 2). The original Western blot images are provided in the Supplementary Materials. Under NG, eNOS protein abundance was higher in ADs, MCSs, and Matrigel than in Controls. Under HG, reduced protein levels were observed in MCSs and Matrigel, whereas ADs retained relatively high expression. In addition to differences in eNOS immunoreactivity, confocal microscopy revealed evident morphological differences among the endothelial models, with visibly larger DAPI-stained nuclei in Control and adherent clinorotated cells than in MCS and Matrigel-derived structures. eNOS immunoreactivity was detectable in all groups, with visible differences in signal intensity and intracellular distribution.

### 2.2. PI3K CA, AKT1, and mTOR Expression

**Figure 3:**
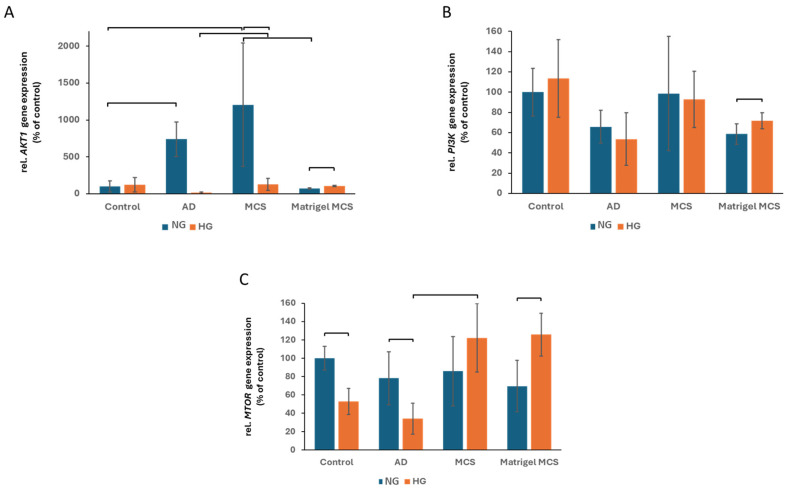
*PI3K CA-AKT-mTOR*-related gene expression in EA.hy926 endothelial cells cultured under normoglycemic and high-glucose conditions. Relative mRNA expression of (**A**) *AKT1*, (**B**) *PI3K CA*, and (**C**) *mTOR* in static monolayers (Controls), adherent clinorotated cells (ADs), multicellular spheroids (MCSs), and Matrigel-derived endothelial structures after 7 days of culture under normoglycemic conditions (NG, 5.5 mM) or high-glucose conditions (HG, 25 mM). Gene expression was analyzed by qPCR, normalized to 18S rRNA, and expressed relative to the NG Control group using the ΔΔCt method. Blue bars indicate NG, and orange bars indicate HG. Data are presented as mean ± SD from *n* = 5 independent biological replicates. Overall group differences were analyzed by one-way ANOVA followed by Tukey’s post hoc test. Predefined pairwise comparisons were analyzed using the Mann–Whitney U test. Statistical significance was accepted at *p* < 0.05. Horizontal brackets indicate statistically significant comparisons.

Expression within the *PI3K CA-AKT1-mTOR* axis also varied across the four endothelial models (Figure 3). For *PI3K CA*, no consistent significant differences were observed between the four models under either NG or HG conditions; the only clear significant change was the increase in Matrigel under HG compared with NG. For *AKT1*, the main significant difference was observed between ADs and MCSs. For *mTOR*, expression was reduced under HG in Controls and ADs, whereas no comparable reduction was evident in MCSs or Matrigel.

**Figure 4:**
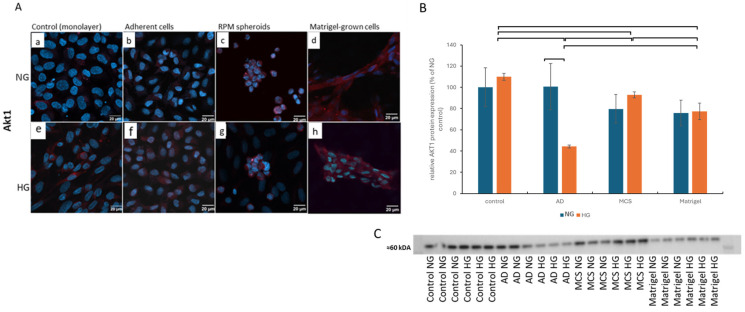
AKT1 protein expression and localization in EA.hy926 endothelial cells cultured under normoglycemic and high-glucose conditions. (**A**) Representative confocal images and (**B**) Western blot analysis of AKT1 in static monolayers (Controls), adherent clinorotated cells (ADs), multicellular spheroids (MCSs), and Matrigel-derived endothelial structures after 7 days under normoglycemic conditions (NG, 5.5 mM) or high-glucose conditions (HG, 25 mM). Confocal images show AKT immunostaining in red and nuclei counterstained with DAPI in blue. Panels (**a**–**d**) represent NG conditions, and panels (**e**–**h**) represent HG conditions: (**a**,**e**) Control; (**b**,**f**) AD; (**c**,**g**) MCS; (**d**,**h**) Matrigel. Scale bar = 20 µm. The bar graph shows densitometric quantification of AKT protein expression normalized to the NG Control group. (**C**) A representative Western blot is shown below. Unlabeled lanes contain protein markers. Data are presented as mean ± SD from *n* = 5 independent biological replicates. Overall group differences were analyzed by one-way ANOVA followed by Tukey’s post hoc test. Predefined pairwise comparisons were analyzed using the Mann–Whitney U test. Statistical significance was accepted at *p* < 0.05. Horizontal brackets indicate statistically significant comparisons.

Protein analysis confirmed AKT1 expression in all groups (Figure 4). Western blot quantification showed only limited differences in AKT protein abundance between most groups; the most evident change was the reduction in ADs under HG compared with NG. In contrast, Controls, MCSs, and Matrigel showed only minor differences between glucose conditions. Confocal microscopy demonstrated AKT immunoreactivity in monolayers, adherent cells, spheroids, and Matrigel-derived structures.

### 2.3. VCAM1 and ICAM1 Expression

**Figure 5:**
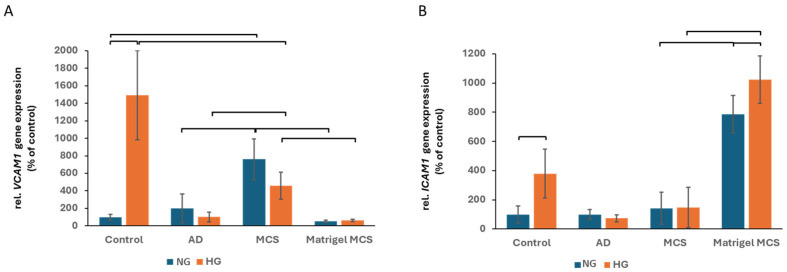
*VCAM1* and *ICAM1* gene expressions in EA.hy926 endothelial cells cultured under normoglycemic and high-glucose conditions. Relative mRNA expression of (**A**) *VCAM1* and (**B**) *ICAM1* in static monolayers (Controls), adherent clinorotated cells (ADs), multicellular spheroids (MCSs), and Matrigel-derived endothelial structures after 7 days of culture under normoglycemic conditions (NG, 5.5 mM) or high-glucose conditions (HG, 25 mM). Gene expression was analyzed by qPCR, normalized to 18S rRNA, and expressed relative to the NG Control group using the ΔΔCt method. Blue bars indicate NG, and orange bars indicate HG. Data are presented as mean ± SD from *n* = 5 independent biological replicates. Overall group differences were analyzed by one-way ANOVA followed by Tukey’s post hoc test. Predefined pairwise comparisons were analyzed using the Mann–Whitney U test. Statistical significance was accepted at *p* < 0.05. Horizontal brackets indicate statistically significant comparisons.

Expression of *VCAM1* and *ICAM1* differed across the four endothelial models (Figure 5). For *VCAM1*, the most pronounced increase was observed in Controls under HG. ADs and Matrigel remained low under both glucose conditions, whereas MCSs showed intermediate expression with lower levels under HG than under NG. For *ICAM1*, the highest expression was observed in Matrigel under both NG and HG, while Controls showed a marked increase under HG compared with NG. In contrast, there were no changes in ICAM1 in ADs and MCSs in NG and HG.

**Figure 6:**
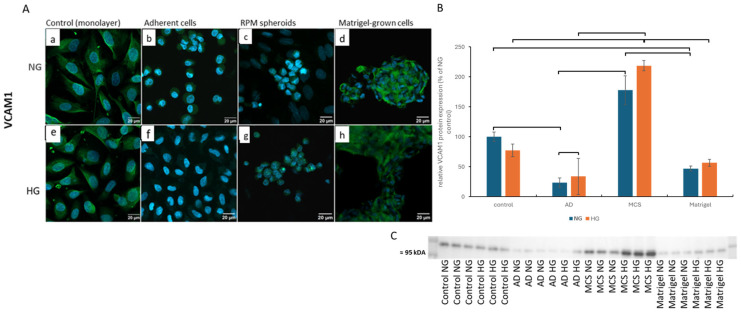
VCAM1 protein expression and localization in EA.hy926 endothelial cells cultured under normoglycemic and high-glucose conditions. (**A**) Representative confocal images and (**B**) Western blot analysis of VCAM1 in static monolayers (Controls), adherent clinorotated cells (ADs), multicellular spheroids (MCSs), and Matrigel-derived endothelial structures after 7 days under normoglycemic conditions (NG, 5.5 mM) or high-glucose conditions (HG, 25 mM). Confocal images show VCAM1 immunostaining in green and nuclei counterstained with DAPI in blue. Panels (**a**–**d**) represent NG conditions, and panels (**e**–**h**) represent HG conditions: (**a**,**e**) Control; (**b**,**f**) AD; (**c**,**g**) MCS; (**d**,**h**) Matrigel. Scale bar = 20 µm. The bar graph shows densitometric quantification of VCAM1 protein expression normalized to the NG Control group. (**C**) A representative Western blot of the different conditions is shown below. Unlabeled lanes contain protein markers. Data are presented as mean ± SD from *n* = 5 independent biological replicates. Overall group differences were analyzed by one-way ANOVA followed by Tukey’s post hoc test. Predefined pairwise comparisons were analyzed using the Mann–Whitney U test. Statistical significance was accepted at *p* < 0.05. Horizontal brackets indicate statistically significant comparisons.

Protein-level analysis confirmed VCAM1 expression in all groups (Figure 6). Confocal microscopy demonstrated VCAM1 immunoreactivity in monolayers, adherent cells, spheroids, and Matrigel-derived structures. Western blot quantification showed the strongest VCAM1 protein signal in MCSs, whereas ADs showed the lowest levels. Controls displayed intermediate protein levels, and Matrigel remained low under both glucose conditions. Compared with transcript data, glucose-dependent differences in VCAM1 appeared less pronounced at the protein level than the model-dependent differences.

### 2.4. VEGF-Related Gene and Protein Expression

**Figure 7:**
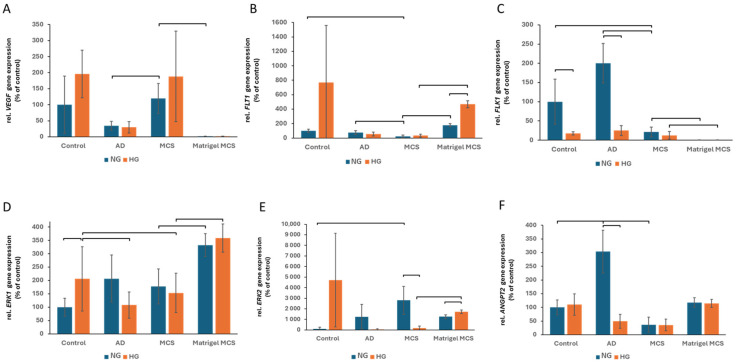
*VEGF*-related angiogenic gene expression in EA.hy926 endothelial cells cultured under normoglycemic and high-glucose conditions. Relative mRNA expression of (**A**) *VEGF*, (**B**) *FLT1*, (**C**) *FLK1* (*VEGFR2*), (**D**) *ERK1*, (**E**) *ERK2*, and (**F**) *ANGPT2* in static monolayers (Controls), adherent clinorotated cells (ADs), multicellular spheroids (MCSs), and Matrigel-grown endothelial structures after 7 days of culture under normoglycemic conditions (NG, 5.5 mM) or high glucose (HG, 25 mM). Gene expression was analyzed by qPCR, normalized to 18S rRNA, and expressed relative to the NG Control group using the ΔΔC_T_ method. Bars represent means ± SD. Blue bars indicate NG, and orange bars indicate HG. Horizontal brackets indicate statistically significant comparisons as shown in the figure. Data are presented as mean ± SD from *n* = 5 independent biological replicates. Overall group differences were analyzed by one-way ANOVA followed by Tukey’s post hoc test. Predefined pairwise comparisons were analyzed using the Mann–Whitney U test. Statistical significance was accepted at *p* < 0.05. Horizontal brackets indicate statistically significant comparisons.

The angiogenesis-related genes displayed distinct model-dependent expression profiles across the four endothelial models (Figure 7). The most pronounced transcript signals were observed for *FLT1* in Controls under HG, *FLK1* (*VEGFR2*) in ADs under NG, *ERK1* in Matrigel under both glucose conditions, *ERK2* in ADs under NG and in Controls under HG, and *ANGPT2* in ADs under NG.

**Figure 8:**
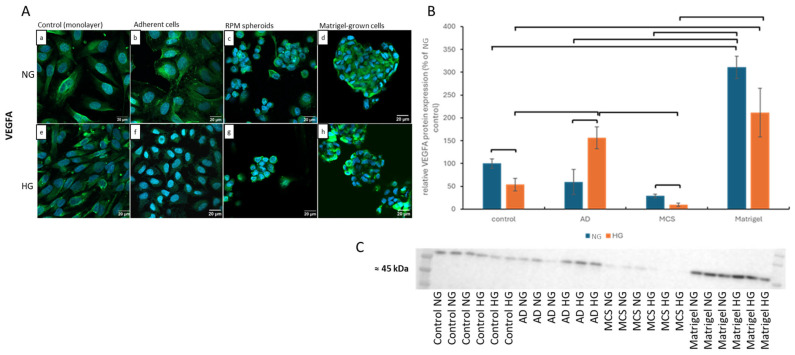
VEGFA protein expression and localization in EA.hy926 endothelial cells cultured under normoglycemic and high-glucose conditions. (**A**) Representative confocal images of *VEGFA* immunostaining in static monolayers (Controls), adherent clinorotated cells (ADs), multicellular spheroids (MCSs), and Matrigel-grown endothelial structures after 7 days under normoglycemic conditions (NG, 5.5 mM) or high glucose (HG, 25 mM). *VEGFA* is shown in green, and nuclei are counterstained with DAPI in blue. Panels (**a**–**d**) represent NG conditions, and panels (**e**–**h**) represent HG conditions: (**a**,**e**) Control; (**b**,**f**) AD; (**c**,**g**) MCS; (**d**,**h**) Matrigel-grown cells. Scale bar = 20 µm. (**B**) Densitometric quantification of VEGFA protein expression measured by Western blot and normalized to the NG Control group. Bars represent mean ± SD. (**C**) Representative Western blot of VEGFA protein. Unlabeled lanes contain protein markers. Data are presented as mean ± SD from *n* = 5 independent biological replicates. Overall group differences were analyzed by one-way ANOVA followed by Tukey’s post hoc test. Predefined pairwise comparisons were analyzed using the Mann–Whitney U test. Statistical significance was accepted at *p* < 0.05. Horizontal brackets indicate statistically significant comparisons.

VEGFA protein was detected in all endothelial models (Figure 8). Confocal microscopy showed VEGFA immunoreactivity in all groups, with a more organized pattern in Matrigel under NG than under HG. Western blot analysis showed the strongest VEGFA-associated protein signal in Matrigel and the weakest in MCSs.

### 2.5. Vinculin- and Junction-Related Gene and Protein Expression

**Figure 9:**
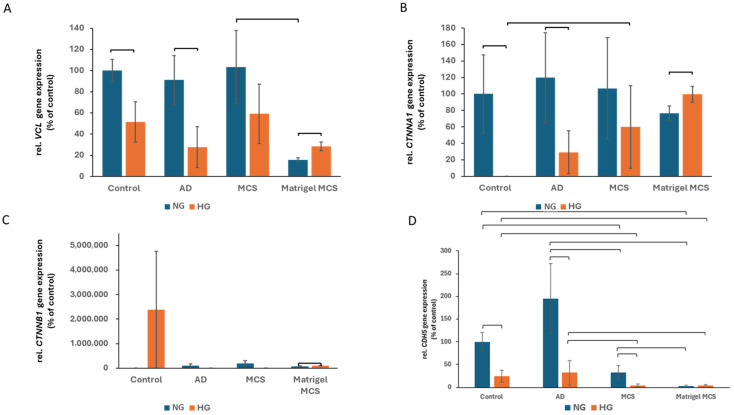
Vinculin- and junction-related gene expression in EA.hy926 endothelial cells cultured under normoglycemic and high-glucose conditions. Relative mRNA expression of (**A**) *VCL*, (**B**) *CTNNA1*, (**C**) *CTNNB1*, and (**D**) *CDH5* in static monolayers (Controls), adherent clinorotated cells (ADs), multicellular spheroids (MCSs), and Matrigel-grown endothelial structures after 7 days of culture under normoglycemic conditions (NG, 5.5 mM) or high-glucose conditions (HG, 25 mM). Transcript levels were analyzed by qPCR, normalized to 18S rRNA, and expressed relative to the NG Control group using the ΔΔCt method. Blue bars represent NG, and orange bars represent HG. Data are presented as mean ± SD from *n* = 5 independent biological replicates. Overall group differences were analyzed by one-way ANOVA followed by Tukey’s post hoc test. Predefined pairwise comparisons were analyzed using the Mann–Whitney U test. Statistical significance was accepted at *p* < 0.05. Horizontal brackets indicate statistically significant pairwise comparisons.

The structural adhesion markers showed distinct model-dependent expression profiles across the four endothelial models (Figure 9). VCL expression was lowest in Matrigel under NG and increased under HG in Matrigel, whereas Controls, ADs, and MCSs showed comparatively higher expression under NG than under HG. CTNNA1 showed the highest expression in ADs and MCSs under NG and was generally reduced under HG, although Matrigel displayed higher expression under HG than under NG. In contrast, CTNNB1 showed a striking increase in Controls under HG, whereas the other models remained substantially lower. CDH5 expression was highest in ADs under NG, with lower levels in Controls and MCSs and minimal expression in Matrigel. Under HG, CDH5 expression was reduced across all models and remained particularly low in MCSs and Matrigel.

**Figure 10:**
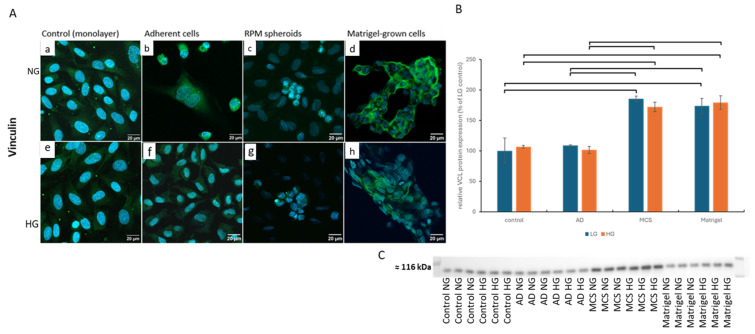
Vinculin protein expression and localization in EA.hy926 endothelial cells cultured under normoglycemic and high-glucose conditions. (**A**) Representative confocal images of vinculin immunostaining in static monolayers (Controls), adherent clinorotated cells (ADs), multicellular spheroids (clinostat-derived spheroids/MCSs), and Matrigel-grown endothelial structures after 7 days under normoglycemic conditions (NG, 5.5 mM) or high glucose (HG, 25 mM). Vinculin is shown in green, and nuclei are counterstained with DAPI in blue. Panels (**a**–**d**) represent NG conditions, and panels (**e**–**h**) represent HG conditions: (**a**,**e**) Control; (**b**,**f**) AD; (**c**,**g**) clinostat-derived spheroids; (**d**,**h**) Matrigel-grown cells. Scale bar = 20 µm. (**B**) Densitometric quantification of vinculin protein expression measured by Western blot and normalized to the NG Control group. Bars represent mean ± SD. (**C**) Representative Western blot of vinculin. Unlabeled lanes contain protein markers. Data are presented as mean ± SD from *n* = 5 independent biological replicates. Overall group differences were analyzed by one-way ANOVA followed by Tukey’s post hoc test. Predefined pairwise comparisons were analyzed using the Mann–Whitney U test. Statistical significance was accepted at *p* < 0.05. Horizontal brackets indicate statistically significant comparisons.

Vinculin protein was detected in all endothelial models (Figure 10). Confocal microscopy demonstrated vinculin immunoreactivity in monolayers, adherent cells, spheroids, and Matrigel-derived structures, with stronger staining in MCSs and Matrigel than in Controls and ADs. Western blot quantification showed the highest vinculin protein levels in MCSs and Matrigel, whereas Controls and ADs had lower levels. Differences between NG and HG were less pronounced at the protein level than at the transcript level.

**Figure 11:**
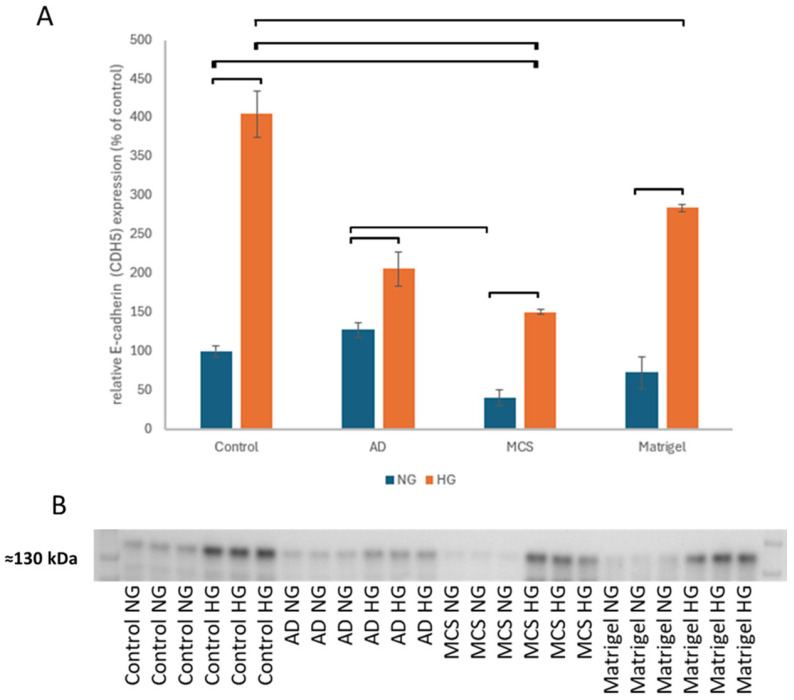
CDH5/VE-cadherin protein expression in EA.hy926 endothelial cells cultured under normoglycemic and high-glucose conditions. (**A**) Densitometric quantification of CDH5/VE-cadherin protein expression in static monolayers (Controls), adherent clinorotated cells (ADs), multicellular spheroids (MCSs), and Matrigel-derived endothelial structures after 7 days under normoglycemic conditions (NG, 5.5 mM) or high glucose (HG, 25 mM). Protein expression was determined by Western blot and normalized to the NG Control group. Blue bars indicate NG, and orange bars indicate HG. (**B**) Representative Western blot of CDH5/VE-cadherin protein expression. Unlabeled lanes contain protein markers. Data are presented as mean ± SD from *n* = 5 independent biological replicates. Overall group differences were analyzed by one-way ANOVA followed by Tukey’s post hoc test. Predefined pairwise comparisons were analyzed using the Mann–Whitney U test. Statistical significance was accepted at *p* < 0.05. Horizontal brackets indicate statistically significant comparisons.

Additional CDH5 protein analysis showed marked model- and glucose-dependent differences (Figure 11). CDH5 protein abundance was increased under HG in all four models, with the strongest signal in HG Controls and HG Matrigel, intermediate levels in HG ADs, and the lowest levels in HG MCSs. Under NG, the CDH5 protein was highest in ADs, lower in Controls and Matrigel, and lowest in MCSs. Thus, unlike the transcript data, CDH5 protein expression showed a consistent increase under HG across all models, indicating discordance between junction-related transcriptional and protein-level responses.

## 3. Discussion

The present study shows that endothelial responses to hyperglycemia are not fixed properties of the cell line alone but are strongly shaped by growth configuration, mechanical loading, and matrix context. Across the four models, high glucose did not induce a uniform phenotype; instead, it differentially altered nitric oxide-related signaling, PI3K CA-AKT-mTOR signaling, inflammatory adhesion, angiogenic organization, and structural adhesion. These are important points because diabetic endothelial dysfunction is now understood as a multidimensional process involving impaired NO bioavailability, altered mechanotransduction, inflammatory activation, and remodeling of angiogenic and barrier-related pathways rather than a single linear defect [9,10,11,12]. The divergence between adherent clinorotated cells and multicellular spheroids likely reflects, at least in part, the transition from substrate-attached endothelial growth to non-adherent three-dimensional aggregation. This shift is expected to alter cell–matrix signaling, cell–cell packing, force transmission, and local microenvironmental conditions, all of which may modify endothelial responses to glucose stress. Although the present design does not isolate each variable, the findings support the conclusion that endothelial phenotype in this system is strongly shaped by structural state rather than by glucose exposure alone [18,23].

A central observation of the present study was the downregulation of *eNOS*-related transcription under high glucose conditions in most models, with the strongest reductions in the spheroid and clinorotated populations, whereas adherent clinorotated cells retained comparatively strong eNOS protein expression. This pattern is consistent with current concepts of diabetic endothelial dysfunction, in which impaired NO signaling is driven not only by reduced eNOS expression or activity but also by oxidative stress, uncoupling, and diminished NO responsiveness at the vascular level [9,10,11]. NOSIP is an eNOS-interacting regulatory protein that promotes redistribution of eNOS away from the plasma membrane and thereby modulates nitric oxide production [32]. The parallel decrease in NOSIP in Controls, ADs, and MCSs suggests that hyperglycemia affected not only the NO-producing enzyme axis itself but also its regulatory environment. The divergent Matrigel pattern, with relative preservation of eNOS-related transcripts but lower protein levels under HG, suggests that extracellular matrix-guided organization can buffer transcriptional loss while still remaining vulnerable at the protein or architectural level.

The PI3K CA-AKT-mTOR data further support the view that hyperglycemia rewires endothelial signaling in a context-dependent manner rather than simply suppressing it globally. PI3K CA was strongly induced in ADs and MCSs under NG and collapsed under HG, whereas AKT1 was comparatively more stable, and mTOR showed the opposite pattern of regulation in different models. This separation between upstream and downstream nodes of the pathway is biologically plausible. Recent mechanotransduction literature indicates that endothelial force sensing is distributed across junctional complexes, ion channels, the glycocalyx, the cytoskeleton, and membrane-associated signaling hubs, with PI3K CA-AKT signaling serving as a major integrator of shear-sensitive responses [2,5,6,25,26]. In that framework, the strong PI3K CA response in clinostat-derived conditions under NG likely reflects active force adaptation, whereas its suppression under HG suggests that hyperglycemia compromises this adaptive signaling state. The relative preservation of AKT protein in MCSs and Matrigel, despite transcriptional divergence, also indicates that post-transcriptional stabilization and pathway branching should be considered when interpreting endothelial stress adaptation.

The inflammatory adhesion profile also differed clearly between models. VCAM1 was highest in MCSs under NG and remained prominent at the protein level, whereas HG caused the strongest transcriptional increase in static monolayers. This suggests that the spheroid condition may represent a structurally pre-activated endothelial state, while the monolayer condition more strongly captures an acute glucose-driven inflammatory response. By contrast, ICAM1 was most pronounced in Matrigel, particularly under HG, indicating that matrix-guided endothelial organization does not necessarily represent a quiescent phenotype but may instead support a distinct adhesion program. This interpretation is consistent with recent work showing that hyperglycemia promotes endothelial activation through inflammatory and stress-responsive signaling pathways that converge on the expression of adhesion molecules, including VCAM1 and ICAM1 [12]. The incomplete concordance between transcript and protein readouts is not unexpected across structurally distinct endothelial states. Differences in translation, protein turnover, intracellular trafficking, and stabilization may all contribute; transcript and protein data should therefore be interpreted here as complementary rather than interchangeable layers of endothelial adaptation. This is particularly relevant in the present study, where the endothelial models differ not only in glucose exposure but also in substrate attachment, three-dimensional aggregation, and matrix-guided organization, each of which may independently influence protein localization, turnover, and stability.

Collectively, these findings indicate that endothelial responses to hyperglycemia cannot be reduced to a single inflammatory or metabolic pathway. Rather, glucose exposure interacts with structural organization to alter multiple interconnected processes, including inflammatory activation, nitric oxide signaling, angiogenic regulation, and junctional adaptation. The VEGF-related findings provide a further example of this context dependence.

An important limitation applies to interpreting VEGFA protein results in Matrigel cultures. Matrigel is a murine basement membrane extract with a complex and incompletely defined composition that retains biologically active growth factors, which can influence cellular behavior and complicate the interpretation of matrix-associated signaling [19,20,21]. Accordingly, the VEGFA protein signal detected in Matrigel-grown cultures cannot be assigned unequivocally to endothelial cell-derived VEGFA alone. This interpretation is supported by the very low VEGFA mRNA expression observed in Matrigel-grown cells, consistent with feedback regulation in the presence of exogenous matrix-associated growth factor cues. Therefore, the VEGFA protein findings in Matrigel should be interpreted with caution and considered primarily in the context of angiogenic organization and VEGFA-associated signaling distribution rather than as a direct measure of endothelial VEGFA production.

Within this limitation, the transcript-level data remain informative. FLT1 (VEGFR1) is generally considered a modulatory VEGF receptor with weaker kinase activity than VEGFR2 and can regulate VEGF bioavailability through ligand binding. The strong FLT1 expression observed in HG Control cultures may therefore reflect an adaptive response limiting VEGFR2-driven angiogenic signaling under hyperglycemic stress. In contrast, the reduction in FLK1/VEGFR2 observed in several HG conditions is consistent with attenuation of canonical pro-angiogenic signaling. Together, these findings suggest that hyperglycemia may alter not only the magnitude of VEGF signaling but also the balance between FLT1/VEGFR1- and FLK1/VEGFR2-associated receptor pathways. The strong ERK1 signal in Matrigel and the heterogeneous ERK2 pattern across models further support the concept that endothelial angiogenesis is governed by the interplay among receptor signaling, matrix anchorage, cellular mechanics, and collective organization [25,26,27,28]. The vinculin- and junction-related data add a further structural dimension to these findings. Vinculin protein was highest in MCSs and Matrigel, whereas the transcriptional behavior of VCL, CTNNA1, CTNNB1, and CDH5 was heterogeneous and strongly model-dependent. This result is conceptually important. Vinculin was selected here as a mechanosensitive structural adhesion-associated protein that links adhesion complexes to the actin cytoskeleton and reflects force-dependent endothelial adaptation. We acknowledge, however, that integrin-specific analyses would provide more direct mechanistic information on endothelial substrate adhesion and represent an important direction for future work [29,30,31]. Recent work has emphasized that vinculin recruitment is linked to junctional reinforcement, barrier integrity, and mechanically stabilized endothelial architecture [3,29,30,31,33]. The strong vinculin signal in MCSs and Matrigel, therefore, is consistent with the idea that three-dimensional aggregation and matrix-guided organization impose greater structural demands on the adhesion machinery than flat monolayers. In addition, CDH5 showed one of the clearest examples of transcript–protein discordance in the present study (Figure 9 and Figure 11). Whereas CDH5 transcript expression generally decreased under HG conditions, CDH5 protein abundance increased across all four models. This suggests that endothelial junctional integrity may be regulated at multiple levels beyond transcription alone and highlights the importance of combining gene and protein analyses when evaluating endothelial adaptation. Similar discrepancies were also observed for several other markers in the present study, indicating that post-transcriptional regulation, protein stabilization, turnover, and cellular architecture contribute substantially to endothelial phenotype under different growth conditions [3,29,30,31,33]. The weaker glucose dependence observed at the protein level compared with the transcript level further suggests that endothelial architecture may be preserved or reinforced even when individual junctional transcripts fluctuate substantially [3,29,30,31,33].

Across several markers, transcript and protein responses were not fully concordant. This was evident for eNOS, where adherent clinorotated cells retained relatively strong protein expression despite reduced transcript abundance under HG; for AKT1, where protein abundance remained comparatively stable despite marked variation in PI3KCA and mTOR transcript expression; for VCAM1, where the strongest protein signal was observed in MCSs despite the greatest HG-induced transcript increase occurring in static monolayers; for VEGFA, where Matrigel-derived cultures displayed strong protein signal despite low VEGFA transcript expression; and for CDH5, where protein abundance increased under HG despite reduced transcript expression. These observations suggest that post-transcriptional regulation, protein turnover, stabilization, trafficking, and model-specific structural organization contribute substantially to endothelial phenotype.

A distinguishing feature of the present work compared with previous clinostat-based endothelial studies is the simultaneous comparison of multiple endothelial organizational states rather than the evaluation of a single clinostat condition in isolation. Previous studies have largely focused on the biological consequences of simulated microgravity itself [16,22,23,24], whereas the present study demonstrates that endothelial responses to hyperglycemia depend strongly on whether cells remain substrate-attached, form multicellular spheroids, or organize within a matrix-supported angiogenic environment. The findings, therefore, extend the utility of clinostat-derived models beyond microgravity research and provide a practical framework for selecting endothelial culture systems according to the biological process under investigation.

The principal value of comparing these models lies in identifying which aspects of hyperglycemia-associated endothelial dysfunction are preferentially represented in each system [14,18,23,24]. Static monolayers displayed the strongest glucose-dependent inflammatory transcriptional responses and may therefore be useful for studying endothelial activation. Adherent clinorotated cells retained comparatively robust eNOS and AKT protein expression and may be most informative for investigating mechanotransduction-associated adaptation. Multicellular spheroids exhibited prominent VCAM1 and vinculin protein expression, suggesting enhanced structural adaptation and cell–cell interaction effects. Matrigel-derived cultures most clearly revealed angiogenic organization and VEGFA-related responses, although interpretation should remain cautious because Matrigel contains matrix-associated growth-factor cues. Collectively, these observations indicate that no single model captures the full spectrum of hyperglycemia-associated endothelial dysfunction, and that model selection should be guided by the specific biological process under investigation [14,18,23,24].

Taken together, these findings demonstrate that endothelial responses to hyperglycemia emerge from the interaction between metabolic stress and physical context. The complementary use of monolayer cultures, adherent clinorotated cells, multicellular spheroids, and Matrigel-derived structures therefore provides a broader framework for investigating endothelial dysfunction than any single model alone [18,23,24,25,26,27,28,29,30,31,32,33,34,35,36,37].

A limitation of the present work is that it was performed in the immortalized EA.hy926 endothelial cell line; interpretation should therefore consider cell-line-specific features of this model when extrapolating findings to primary endothelial cells [38]. The study also focused on defined transcriptional, protein, and imaging endpoints after 7 days of exposure. Although this design allowed direct comparison across models, it does not fully capture the heterogeneity of primary endothelial beds or the temporal evolution of endothelial dysfunction. Future work should therefore extend these findings to primary endothelial cells, integrate functional readouts such as NO bioavailability and permeability, and combine the present model systems with more advanced flow-based vascular platforms [34,35,36,37]. Such approaches would help distinguish which abnormalities are predominantly driven by hyperglycemia, by altered mechanical loading, or by their interaction.

## 4. Materials and Methods

### 4.1. Study Design

This study investigated how a physiological/normoglycemic glucose condition (NG, 5.5 mM) and a markedly hyperglycemic condition (HG, 25 mM) influence endothelial phenotype across four frequently used in vitro models of EA.hy926 endothelial cells (ECs): static monolayers (1 *g*), adherent cells exposed to clinostat rotation (ADs), multicellular spheroids generated under simulated microgravity (MCSs), and Matrigel-induced angiogenic structures. The aim was to compare the effects of mechanical stimulation, 3D organization, and extracellular matrix (ECM) guidance on endothelial responses to metabolic stress. All cultures were maintained for seven days under NG or HG conditions. Adherent and MCS cultures were established using a 3D clinostat, following the validated methodology described by Jokšienė et al. [24], which applies continuous rotation to average the gravitational vector, thereby approximating microgravity-like conditions. Matrigel structures grew statically at 1 *g*. Five biological replicates were included per condition.

### 4.2. Cell Culture

EA.hy926 endothelial cells (EC; ATCC CRL-2922) were cultured in Dulbecco’s Modified Eagle Medium (DMEM; D4947, Merck, Darmstadt, Germany) supplemented with 10% fetal bovine serum (F7524, Merck), 2 mM L-glutamine (REF: 35050-038; Thermo Fisher Scientific, Waltham, MA, USA), and 1% penicillin-streptomycin (#1570-063; Thermo Fisher Scientific). To ensure a stable cell line, we regularly tested for mycoplasma, and only cells below passage 10 were used. For the experiments, the medium was adjusted to either 5.5 mM glucose (normoglycemic conditions, NG) or 25 mM glucose (high glucose, HG) using D-(+)-glucose solution (G8769, Merck). Cells were incubated at 37 °C in a humidified atmosphere containing 5% CO_2_.

Static monolayers were seeded into standard tissue culture vessels and cultured under NG or HG conditions for seven days. For the 1 *g* condition, 12 T25 cm^2^ cell culture flasks (REF: 83.3910.002; Sarstedt, Nümbrecht, Germany) were seeded with 1 × 10^6^ cells each, filled to capacity without air bubbles, and kept in the incubator for 7 days under either NG or HG conditions. In parallel, the same number of culture flasks was placed on the 3D clinostat and rotated for seven days. During clinorotation, one fraction of the ECs remained adherent and was exposed to continuously changing shear-like mechanical forces. Another fraction of the ECs formed multicellular spheroids (MCSs) during the same seven-day clinorotation period. Under simulated microgravity generated by the 3D clinostat, non-adherent cells spontaneously aggregated into compact spheroids, as previously described [24]. The spheroids were collected by gentle sedimentation and washed with PBS (#14190144, Thermo Fisher Scientific).

For Matrigel-induced structures, growth factor-reduced Matrigel (REF: 356230; Corning™ Matrigel™ GFR Basement Membrane Matrix, New York, NY, USA) was polymerized at 37 °C for 30 min. EA.hy926 cells were seeded onto the gel in 6-well plates at a density of 5 × 10^5^ cells per well and maintained under NG or HG conditions at 1 *g* for up to seven days. Network morphology was evaluated on days 1, 4, and 7 [21].

For immunofluorescence staining, slide flasks (#170920; Thermo Fisher Scientific) were seeded with 5 × 10^4^ cells and used for 1 *g*, clinorotation, and Matrigel culture experiments. Five biological replicates were prepared for each experimental condition.

### 4.3. Gene Expression Analysis

Total RNA was isolated from 1 *g* monolayers, AD cultures, MCSs, and Matrigel structures using an RNeasy Mini Kit (Qiagen, Hilden, Germany). RNA concentration and purity were assessed spectrophotometrically. cDNA synthesis was performed using a commercially available reverse transcription kit. Quantitative PCR (qPCR) was carried out using SYBR Green chemistry (Fast SYBR™ Green Master Mix, Thermo Fisher Scientific, Darmstadt, Germany) targeting key endothelial genes: *eNOS*, *NOSIP*, *ANGPT2*, *CDH5*, *CTNNA1*, *CTNNB1*, *ERK1*, *ERK2*, *FLK1*, *FLT1*, *ICAM1*, *mTOR*, *PI3K CA*, *AKT1*, *VCAM1*, *VCL*, and *VEGFA* (TIB Molbiol, Berlin, Germany). *18S rRNA* served as the reference transcript. Cycling consisted of 95 °C for 20 s followed by 40 cycles of 95 °C for 1 s and 60 °C for 20 s. Product specificity was assessed in a subsequent melting-curve stage. Relative expression was calculated using the ΔΔC_T_ method.

Primer sequences used in this study are listed in Table 1.

### 4.4. Immunoblotting

Protein samples were heated at 95 °C for 5 min before electrophoresis. Equal amounts of total protein (20 µg per lane) were loaded onto Criterion TGX Stain-Free gels (Bio-Rad, Hercules, CA, USA) together with a molecular weight marker. Electrophoresis was performed in running buffer for 30 min at 250 V. After separation, gels were removed from the cassette and exposed to UV light to obtain the total protein image for normalization. PVDF membranes (Bio-Rad, Hercules, CA, USA) were activated in 99.9% ethanol for 5 min and then equilibrated in transfer buffer. Filter papers were also pre-soaked in transfer buffer before assembly of the transfer stack. Proteins were transferred using the Trans-Blot Turbo Transfer System (Bio-Rad, Hercules, CA, USA) for 30 min at 100 V.

Following transfer, membranes were visualized under UV light to confirm total protein transfer and washed in TBST for 10 min. Membranes were then blocked with EveryBlot blocking buffer (Bio-Rad, Hercules, CA, USA) for 5 min and incubated with the respective primary antibodies (Table 2) in blocking buffer for 2 h at room temperature. After four washes in TBST for 10–15 min each, membranes were incubated with HRP-conjugated secondary antibodies diluted in blocking buffer for 2 h at room temperature. After further washing, immunoreactive bands were detected using Clarity Western ECL Substrate (Bio-Rad, Hercules, CA, USA) according to the manufacturer’s instructions. The substrate components were mixed in a 1:1 ratio and applied to the membrane for 5 min in the dark. Chemiluminescent signals were recorded using the Syngene PXi Touch imaging system (Syngene by Synoptics Group), and band intensities were analyzed using Syngene Gene Tools software 4.3.18. Protein expression was normalized to total protein. Antibody details are summarized in Table 2.

### 4.5. Immunohistochemistry/Immunofluorescence

1 *g* monolayers, adherent clinostat cultures, multicellular spheroids, and Matrigel-derived endothelial structures were prepared for immunofluorescence analysis after seven days of culture under normoglycemic or high-glucose conditions with and without clinorotation. Samples were washed with PBS and fixed in 4% paraformaldehyde (REF: 9713.5000, VWR, Darmstadt, Germany) for 15 min at room temperature, as is commonly applied in endothelial imaging studies [23,24]. Following fixation, cells and spheroids were rinsed with PBS, permeabilized with 0.1% Triton X-100 for 10 min, and blocked with 1% bovine serum albumin (BSA; #A9647, Sigma, Taufkirchen, Germany) to minimize non-specific antibody binding. Primary antibodies against eNOS, AKT1, VCAM1, VCL, and VEGFA were used for immunofluorescence at the same dilutions as listed in Table 2. The next day, the samples were washed and incubated with species-appropriate Alexa Fluor-conjugated secondary antibodies (anti-rabbit #4412; anti-mouse #4408) for 1 h at room temperature in the dark. F-actin cytoskeletal structures were visualized using phalloidin (#ab235138), and nuclei were counterstained with DAPI (F6057, Sigma), as routinely applied to assess endothelial morphology and protein localization. For imaging, monolayers and adherent 3D clinostat cultures were examined directly on coverslips, whereas spheroids were transferred onto glass slides prior to imaging. The same procedure was applied to Matrigel-derived structures. Confocal laser scanning microscopy was performed using an LSM 800 Airyscan microscope (Zeiss, Oberkochen, Germany) equipped with 20× and 63× air objectives and laser lines appropriate for each fluorophore. Imaging settings were kept constant across experimental groups to ensure comparability, in accordance with published methodological standards. All confocal images include a 20 µm scale bar to enable accurate spatial comparison and reproducibility.

### 4.6. Statistics

All data are presented as mean ± standard deviation (SD). Statistical analyses were performed using IBM SPSS Statistics 24 software (IBM, Armonk, NY, USA). Potential outliers were identified using Grubbs’ test. Overall differences among multiple groups were evaluated by one-way ANOVA followed by Tukey’s post hoc multiple-comparison test. In addition, predefined biologically relevant pairwise comparisons were analyzed using the Mann–Whitney U test because several datasets were non-normally distributed and the sample size was limited (*n* = 5). Statistical significance was accepted at *p* < 0.05.

## 5. Conclusions

The present study demonstrates that endothelial responses to hyperglycemia are not fixed properties of the cell line alone but are strongly shaped by the structural and mechanical context of the in vitro model. Static monolayers, adherent clinorotated cells, multicellular spheroids, and Matrigel-derived endothelial structures each displayed distinct patterns of gene and protein expression, indicating that no single model captures all relevant aspects of diabetic endothelial dysfunction.

High glucose altered eNOS-related signaling, PI3K CA-AKT-mTOR-associated responses, inflammatory adhesion markers, angiogenesis-related pathways, and structural adhesion proteins in a model-dependent manner. In particular, hyperglycemia reduced eNOS-related expression acress most models, modified PI3K, CA-AKT-mTOR signaling, enhanced inflammatory activation in selected conditions, disrupted angiogenic signaling in Matrigel cultures, and differentially affected vinculin- and junction-related markers. These findings show that endothelial dysfunction in diabetic conditions is not uniform but depends on the interaction between metabolic stress and endothelial architecture.

Taken together, these findings emphasize that endothelial dysfunction in hyperglycemic conditions cannot be fully captured by a single in vitro model. Instead, complementary endothelial systems are required to resolve the distinct effects of metabolic stress on nitric oxide-related signaling, inflammatory activation, angiogenic organization, and structural adhesion. Such an approach may enhance the physiological relevance of experimental models used to investigate diabetic vascular dysfunction.

The present study has several limitations, including the use of a single immortalized endothelial cell line, the absence of physiological laminar-flow controls, and the focus on molecular and imaging endpoints rather than direct functional measurements. Therefore, the findings should be interpreted as comparative model-specific responses rather than a complete representation of vascular pathophysiology.

Despite these limitations, each model provided distinct experimental advantages. Static monolayers most clearly revealed glucose-induced inflammatory activation. Adherent clinorotated cells preserved mechanotransduction-associated signaling and were informative for studying endothelial adaptation to altered mechanical loading. Multicellular spheroids highlighted structural adaptation and cell–cell interaction effects. Matrigel-derived cultures most effectively demonstrated angiogenic organization and VEGF-associated responses, although interpretation is influenced by matrix-derived growth factors. Together, these observations indicate that complementary use of multiple endothelial models provides a broader and more informative framework for studying endothelial dysfunction than any individual model alone.

## Figures and Tables

**Table 1:** Primer sequences used for qPCR analysis.

Gene	Forward Primer	Reverse Primer	Amplicon Length
*eNOS*	CTCGTCCCTGTGGAAAGACAA	ACGATGGTGACTTTGGCTAGCT	84 bp
*NOSIP*	AGAAGCCGTCCCGCAC	CTGTCTAGCGGTGTGAAGTG	91 bp
*AKT1*	CTTCTATGGCGCTGAGATTGTG	CAGCATGAGGTTCTCCAGCTT	94 bp
*VCAM1*	CATGGAATTCGAACCCAAACA	GGCTGACCAAGACGGTTGTATC	86 bp
*VCL*	GTCTCGGCTGCTCGTATCTT	GTCCACCAGCCCTGTCATTT	117 bp
*VEGFA*	CTACCTCCACCATGCCAAGTG	GCGCTGATAGACATCCATGAAC	100 bp
*ANGPT2*	ATGCAGTACAGAACCAGACGG	TCGTGGTCTGATTTAATACTTGGG	120 bp
*CDH5*	GTCCTGCAGATCTCCGCAAT	TGTTGGCCGTGTTATCGTGA	118 bp
*CTNNA1*	AATTTAGCGCTCGCCCAG	ACAAGGGTTGTAACCTGTGTAA	149 bp
*CTNNB1*	GAAACAGCTCGTTGTACCGC	ATCCACTGGTGAACCAAGCA	123 bp
*ERK1*	ACCTGCGACCTTAAGATTTGTGA	AGCCACATACTCCGTCAGGAA	90 bp
*ERK2*	TTCCAACCTGCTGCTCAACA	TCTGTCAGGAACCCTGTGTGAT	102 bp
*FLK1/VEGFR2*	TCTTCTGGCTACTTCTTGTCATCATC	GATGGACAAGTAGCCTGTCTTCAGT	102 bp
*FLT1*	CCCTCGCCGGAAGTTGTAT	GATAATTAACGAGTAGCCACGAGTCAA	83 bp
*ICAM1*	CGGCTGACGTGTGCAGTAAT	CTTCTGAGACCTCTGGCTTCGT	90 bp
*mTOR*	ATCTTGGCCATAGCTAGCCTC	ACAACTGGGTCATTGGAGGG	115 bp
*PI3K CA*	GAGCCCCGAGCGTTTCTG	GGTCGTGGAGGCATTGTTCT	107 bp
*18S rRNA*	GGAGCCTGCGGCTTAATTT	CAACTAAGAACGGCCATGCA	101 bp

**Table 2:** Primary and secondary antibodies used for Western blot and immunofluorescence.

Antibody Type	Target Protein/Secondary Antibody	Host Species/Specificity	Supplier	Catalog Number	Application	Dilution
Primary	eNOS	Rabbit	CST	#9572	WB, IF	1:1000
Primary	VEGFA	Rabbit	Ab	ab46154	WB, IF	1:1000
Primary	AKT1	Rabbit	CST	#9272	WB, IF	1:1000
Primary	VCAM1	Mouse	Ab	ab134047	WB, IF	1:1000
Primary	Vinculin	Mouse	S-A	V9131	WB, IF	1:2000
Primary	CDH5 (VE-Cadherin)	Rabbit	CST	#2500S	WB	1:1000
Secondary	Goat Anti-rabbit (GAR)-HRP Conjugate	Goat anti-rabbit	Bio Rad	#1705046	WB	1:10,000
Secondary	Anti-rabbit IgG (Alexa Fluor 488 Conjugate)	Goat anti-rabbit	CST	#4412	IF	1:10,000
Secondary	Anti-mouse IgG (Alexa Fluor 488 Conjugate)	Goat anti-mouse	CST	#4408	IF	1:10,000
Secondary	Anti-rabbit IgG, HRP-linked	Goat anti-rabbit	CST	#7074	WB	1:1000
Secondary	Anti-mouse IgG, HRP-linked	Horse anti-mouse	CST	#7076	WB	1:1000

WB: Western blot; IF: immunofluorescence; CST: Cell Signaling Technology; Ab: Abcam; S-A: Sigma-Aldrich.

## Data Availability

The data supporting the findings of this study are available from the corresponding author upon reasonable request. The data are not publicly available because they form part of an ongoing research project that will terminate in 2027.

## Supplementary Materials

The following supporting information can be downloaded at: https://www.mdpi.com/article/10.3390/ijms27146233/s1.

## Abbreviations

The following abbreviations are used in this manuscript:

1 *g*	static normogravity condition
AD	adherent clinorotated cells
AKT1	AKT serine/threonine kinase 1
ANGPT2	angiopoietin 2
Ct	cycle threshold
CTNNA1	catenin alpha 1
CTNNB1	catenin beta 1
CDH5/VE-cadherin	Cadherin 5/vascular endothelial cadherin
DAPI	4′,6-diamidino-2-phenylindole
DMEM	Dulbecco’s Modified Eagle Medium
EC	endothelial cells
ERK1/2	extracellular signal-regulated kinase ½
FBS	fetal bovine serum
FLK1/VEGFR2/KDR	fetal liver kinase 1/vascular endothelial growth factor receptor 2/kinase insert domain receptor
FLT1/VEGFR1	fms-related receptor tyrosine kinase 1/vascular endothelial growth factor receptor 1
HG	high glucose
HRP	horseradish peroxidase
ICAM1	intercellular adhesion molecule 1
NG	normoglycemic conditions
MCS	multicellular spheroids
mTOR/MTOR	mechanistic target of rapamycin
NOSIP	nitric oxide synthase interacting protein
PBS	phosphate-buffered saline
PI3K/PIK3CA	phosphoinositide 3-kinase
PVDF	polyvinylidene fluoride
qPCR	quantitative polymerase chain reaction
SD	standard deviation
VEGF/VEGFA	vascular endothelial growth factor/vascular endothelial growth factor A
VCL	Vinculin
VCAM1	vascular cell adhesion molecule 1
WB	Western blot
