# Supplementary figures and images for: Comparative Analysis of Endothelial Cell Culture Models Under Altered Mechanical Conditions and Glucose Variations
Source: Int J Mol Sci. 2026 Jul 13;27(14):6233. doi: 10.3390/ijms27146233 (PMC13410307; doi:10.3390/ijms27146233)

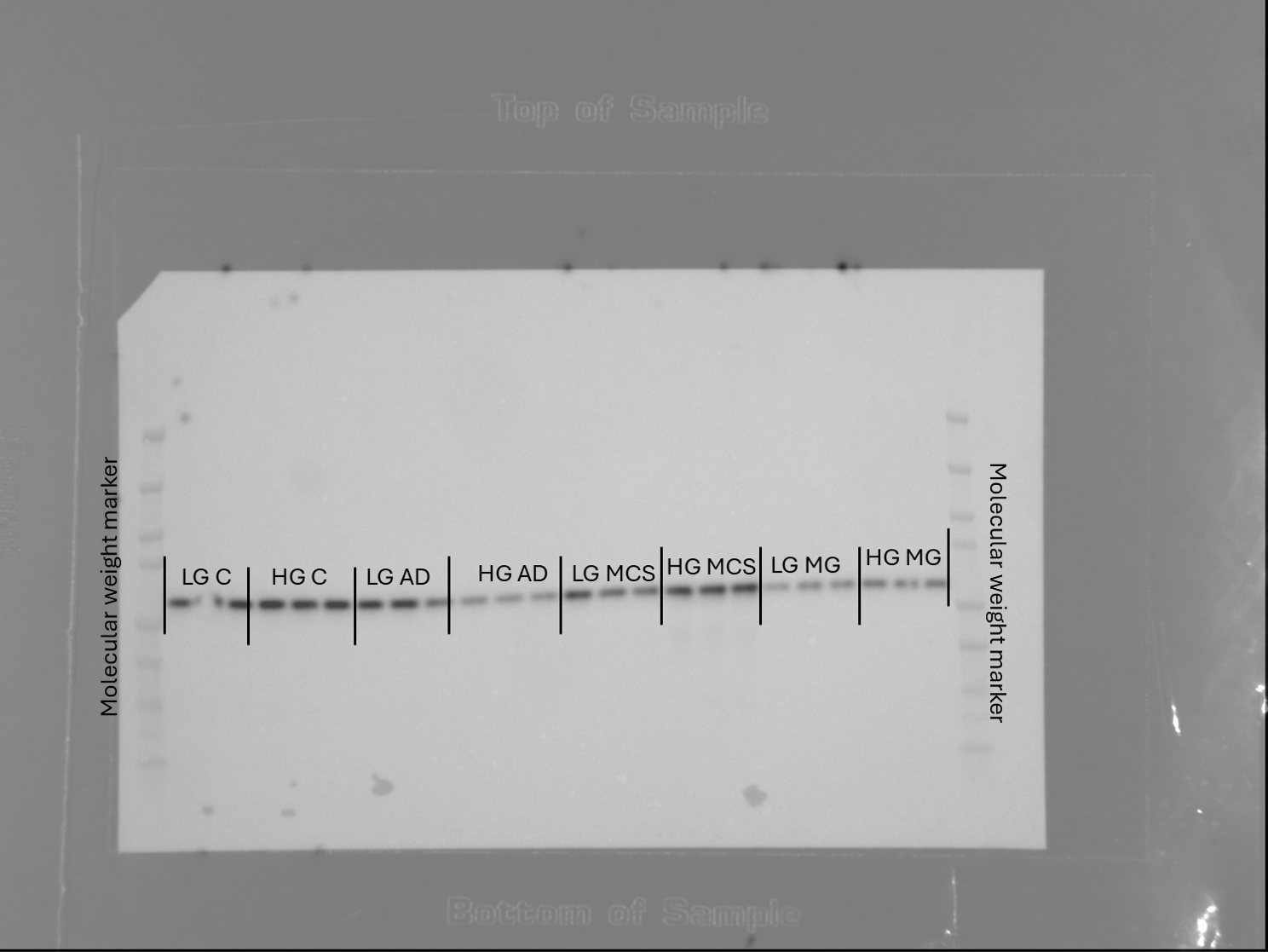

Supplement: Supplementary file 1 [file ijms-27-06233-s001.zip › AKT1 LG_HG combined_figure 4.png]

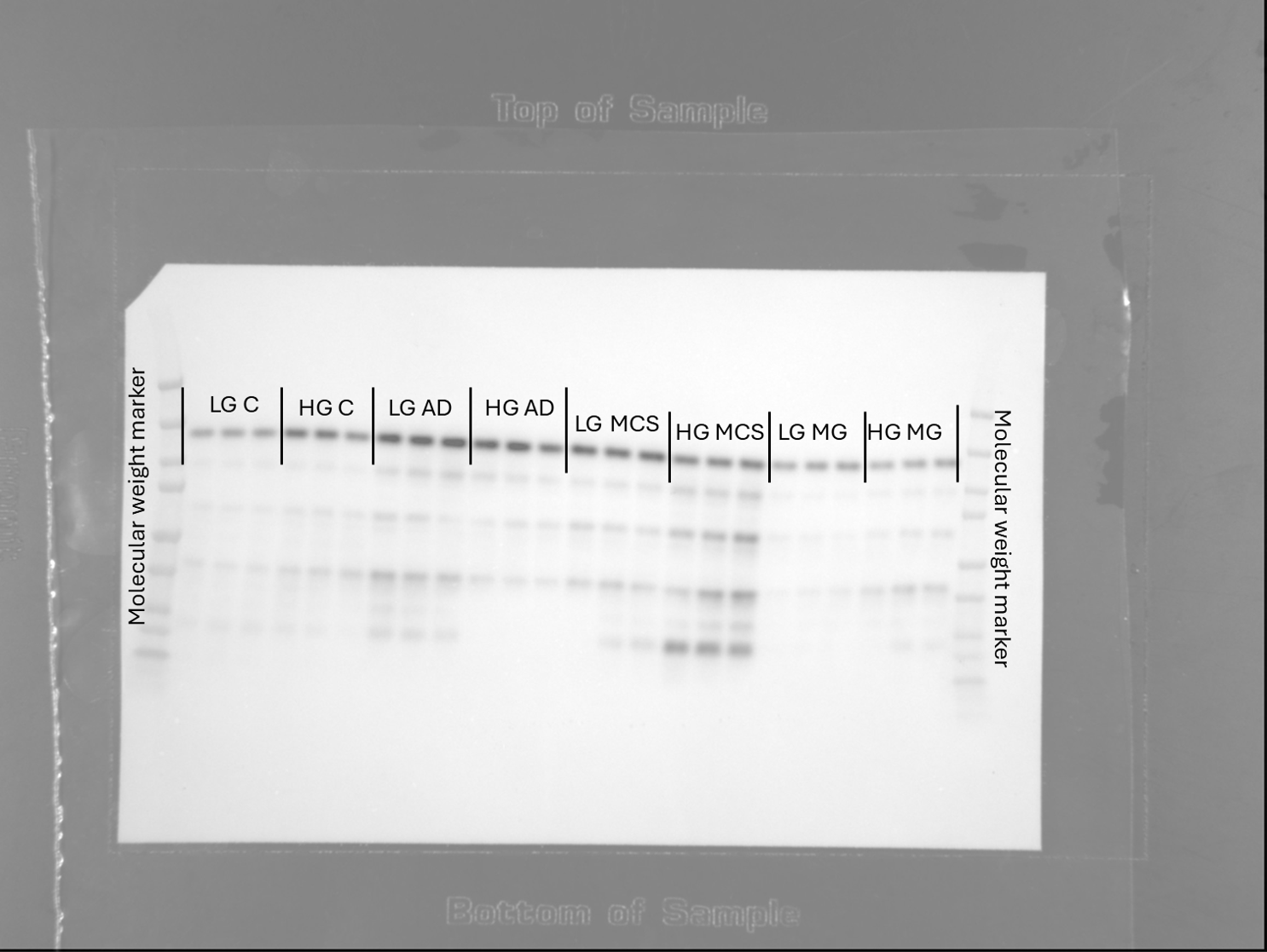

Supplement: Supplementary file 1 [file ijms-27-06233-s001.zip › eNOS LG_HG combined_figure2.png]

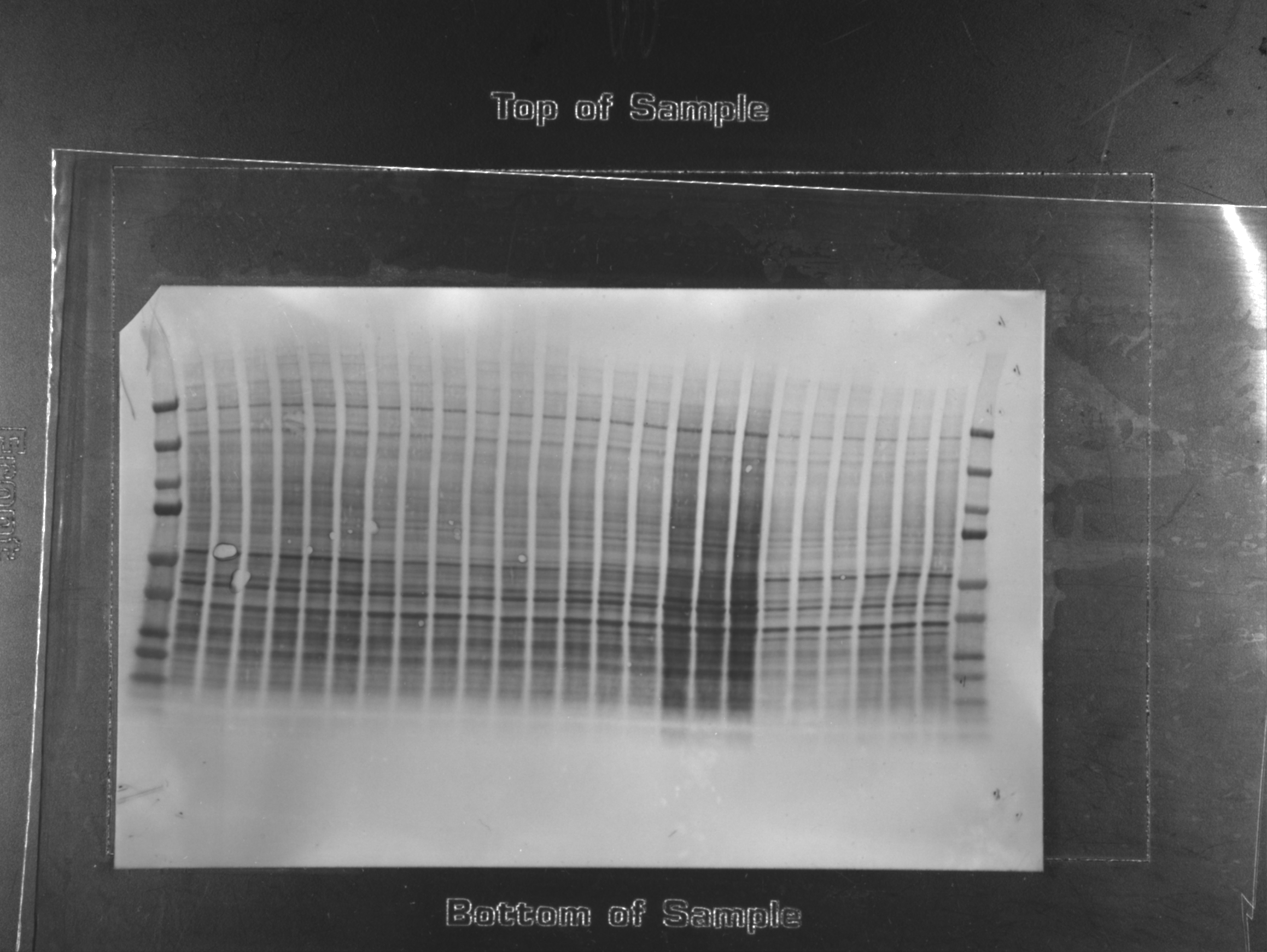

Supplement: Supplementary file 1 [file ijms-27-06233-s001.zip › Total protein ENOS LG HG COMBINED-As-Displayed.png]

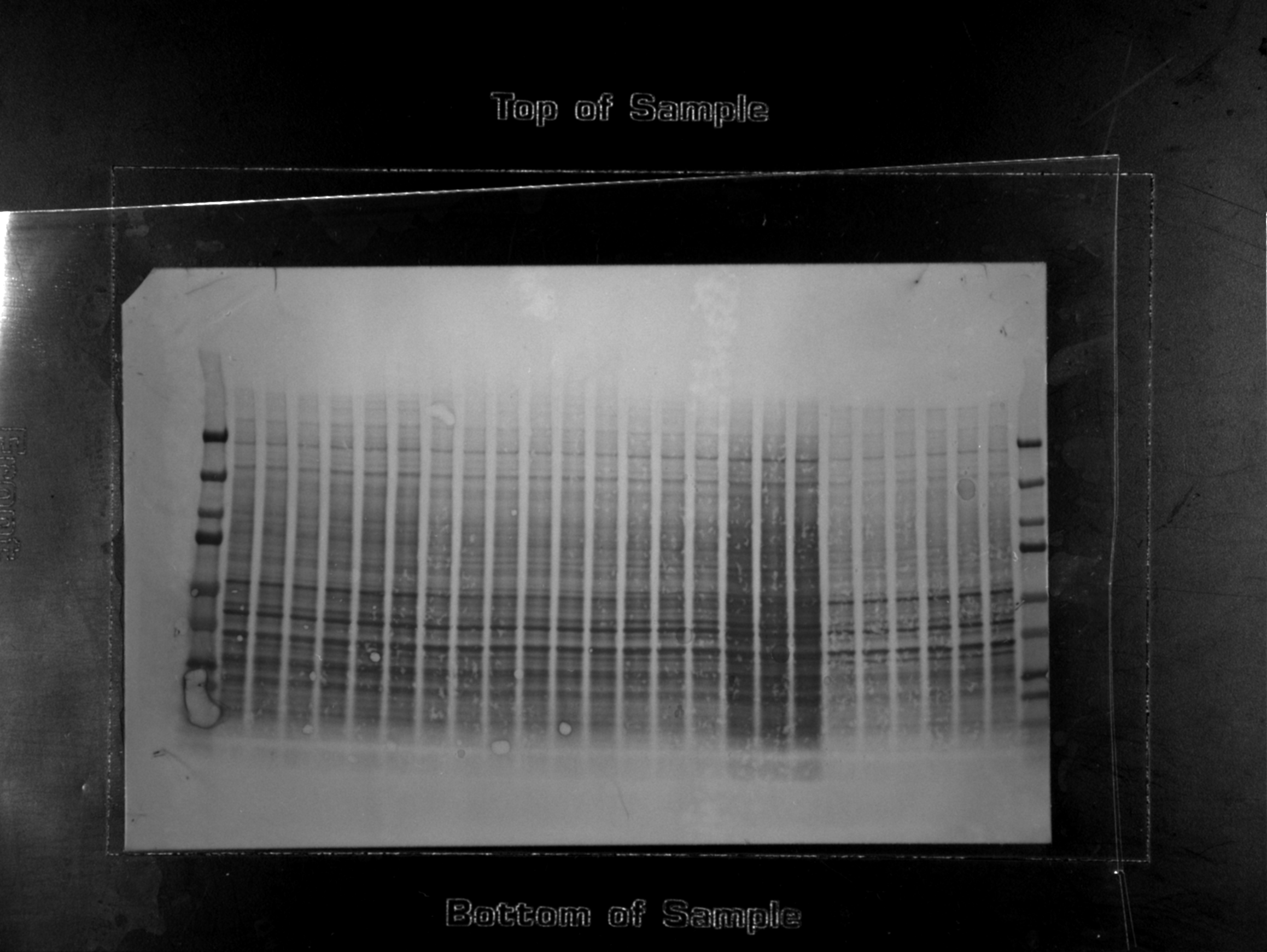

Supplement: Supplementary file 1 [file ijms-27-06233-s001.zip › Total protein VCAM1 CombinedLG HG membrane-As-Displayed(1).png]

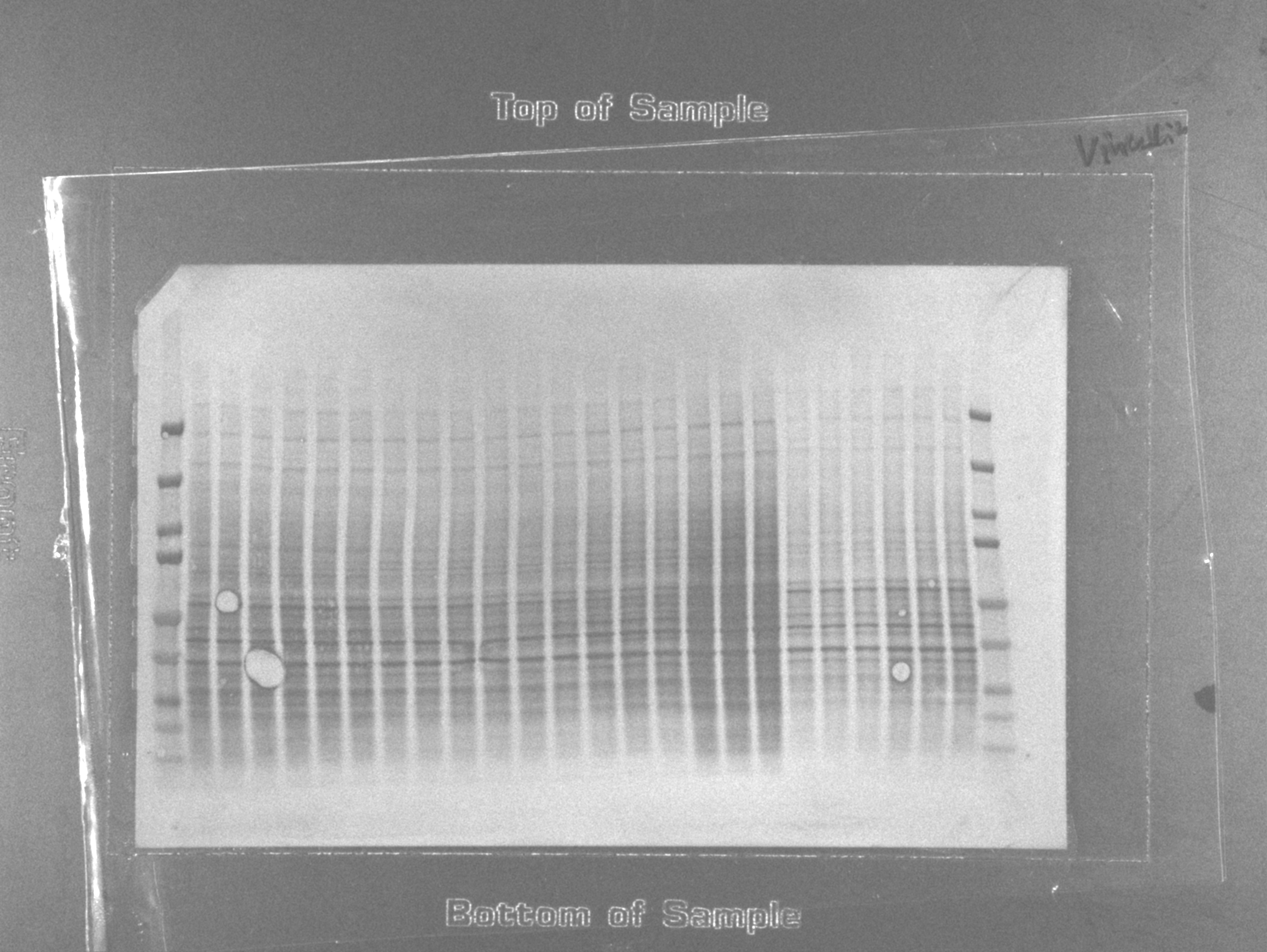

Supplement: Supplementary file 1 [file ijms-27-06233-s001.zip › Total protein vinculin akt membrane combi-As-Displayed.png]

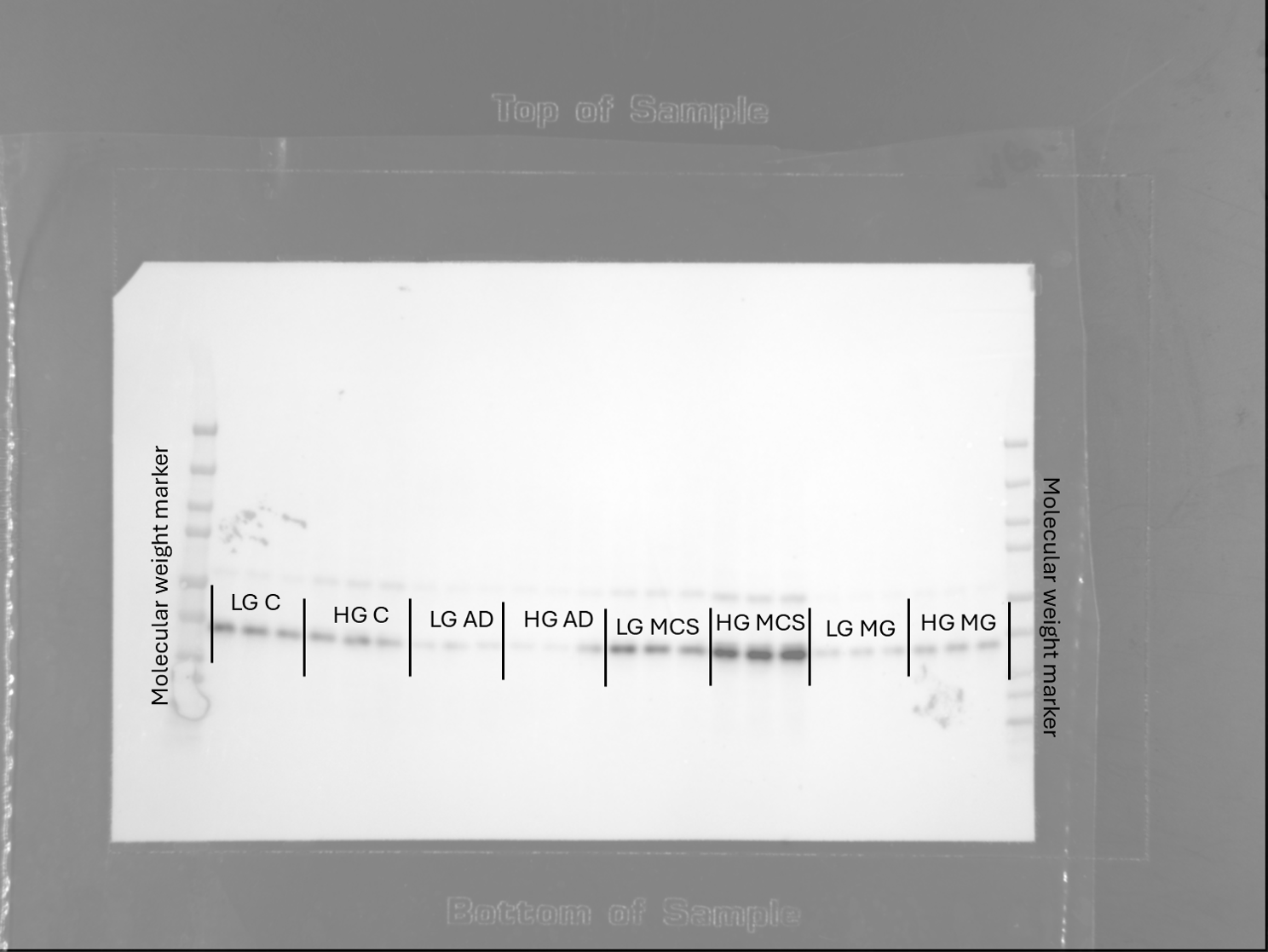

Supplement: Supplementary file 1 [file ijms-27-06233-s001.zip › VCAM1 LG_HG combined_ figure 6.png]

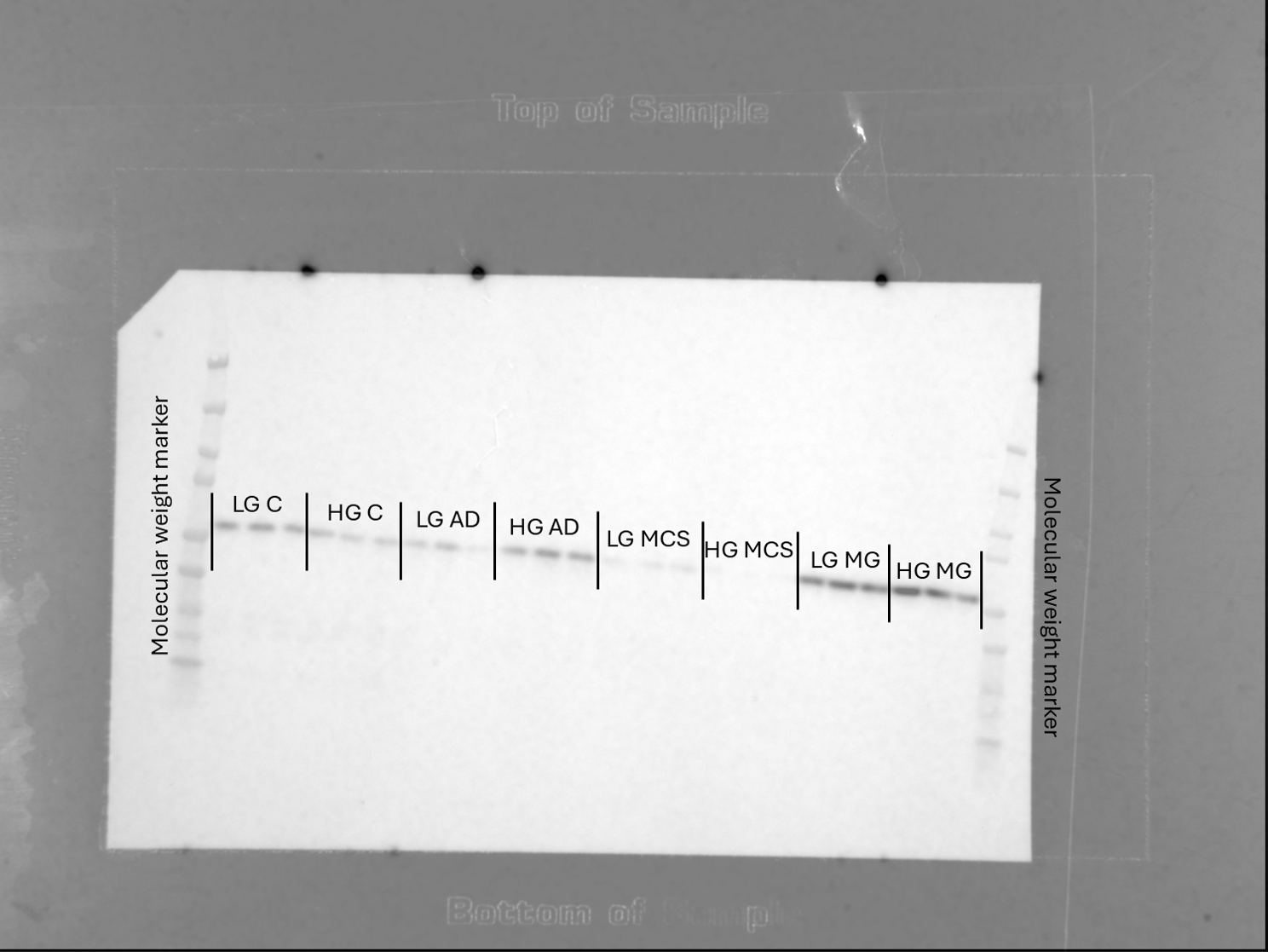

Supplement: Supplementary file 1 [file ijms-27-06233-s001.zip › VEGFA LG_HG combined_ figure 8.png]

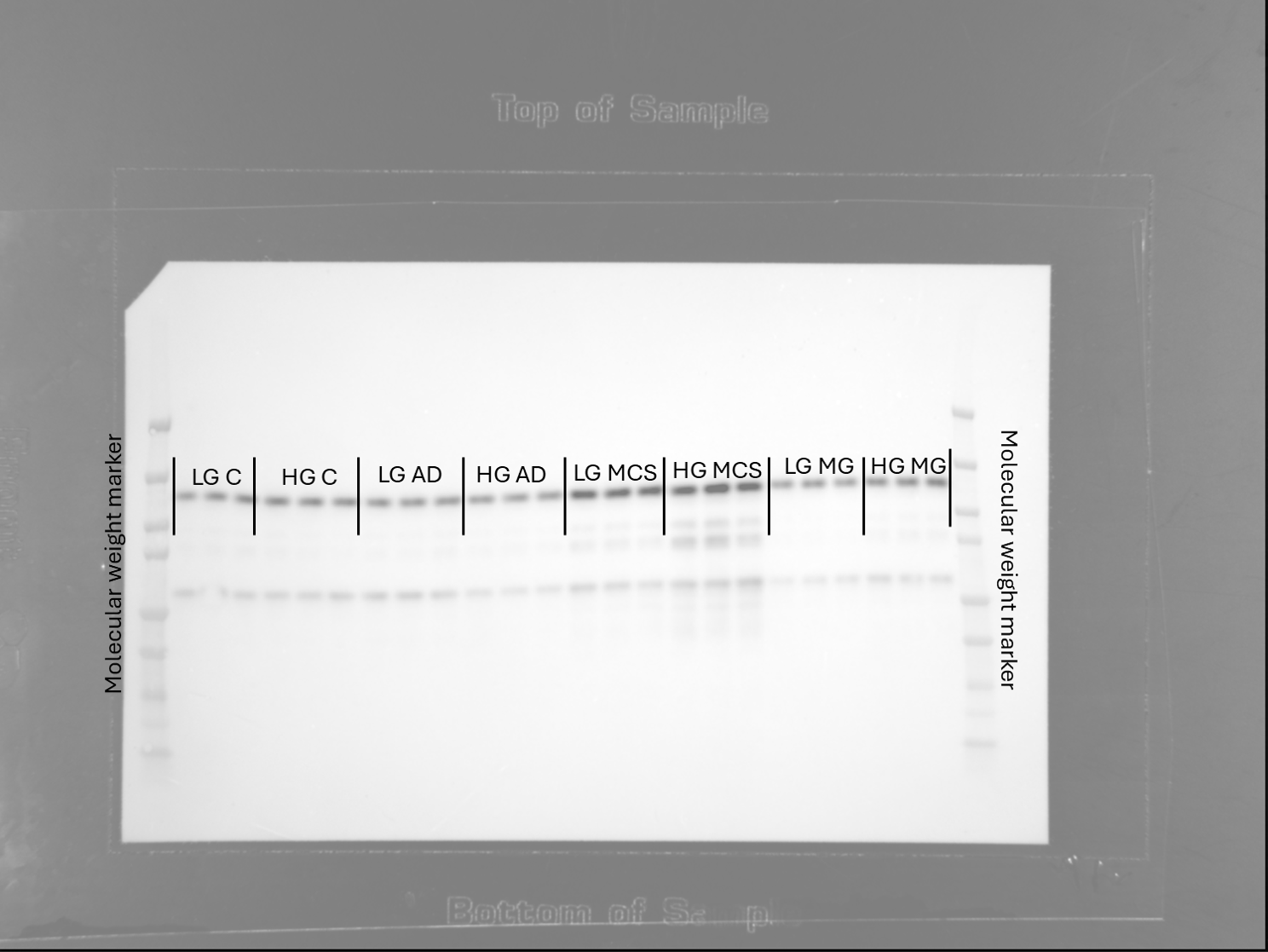

Supplement: Supplementary file 1 [file ijms-27-06233-s001.zip › Vinculin in AKT membrane combined LG_HG_ figure 10.png]

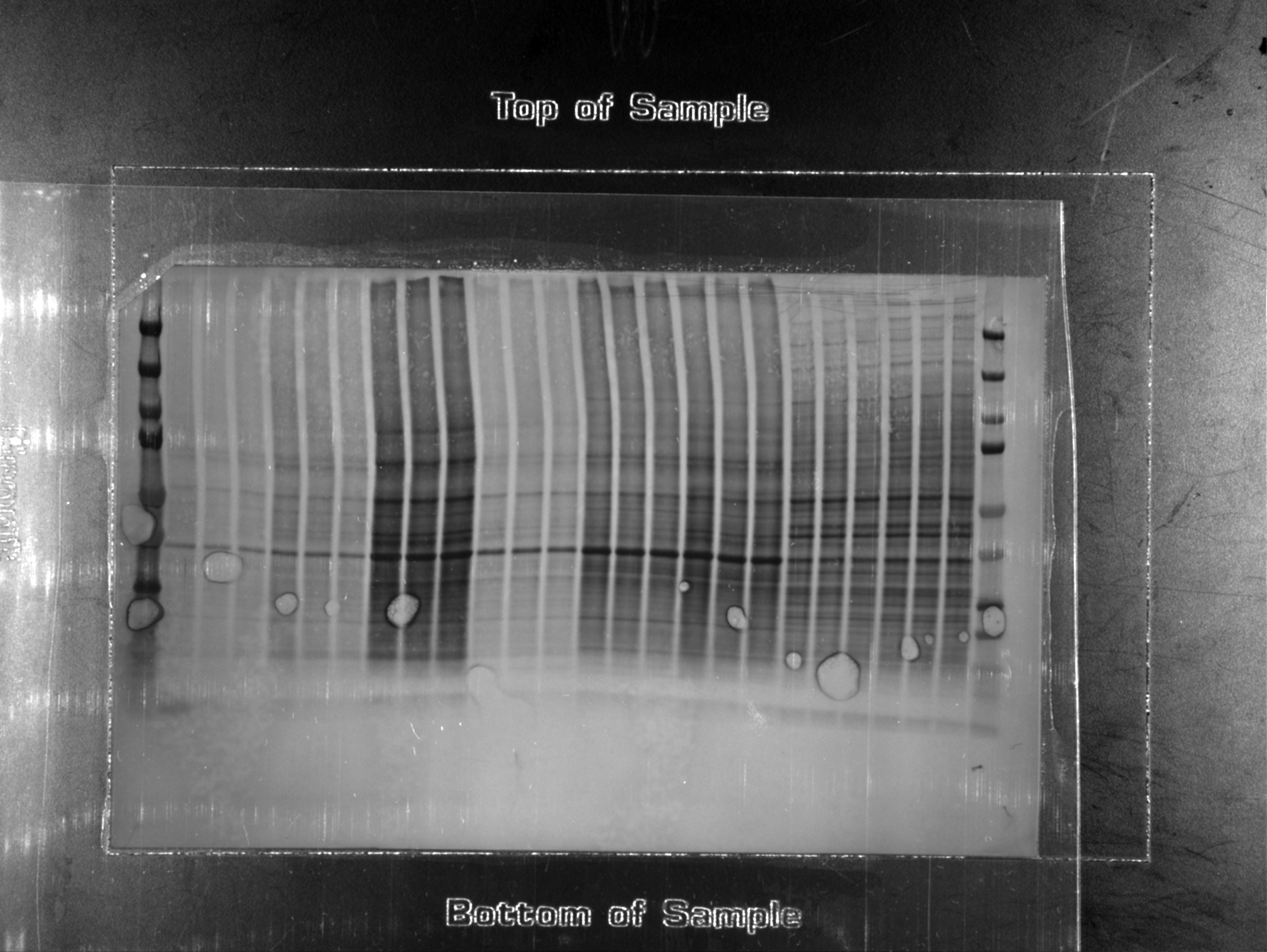

Supplement: Supplementary file 1 [file ijms-27-06233-s001.zip › Total protein combined vinculin-As-Displayed(1).tif]

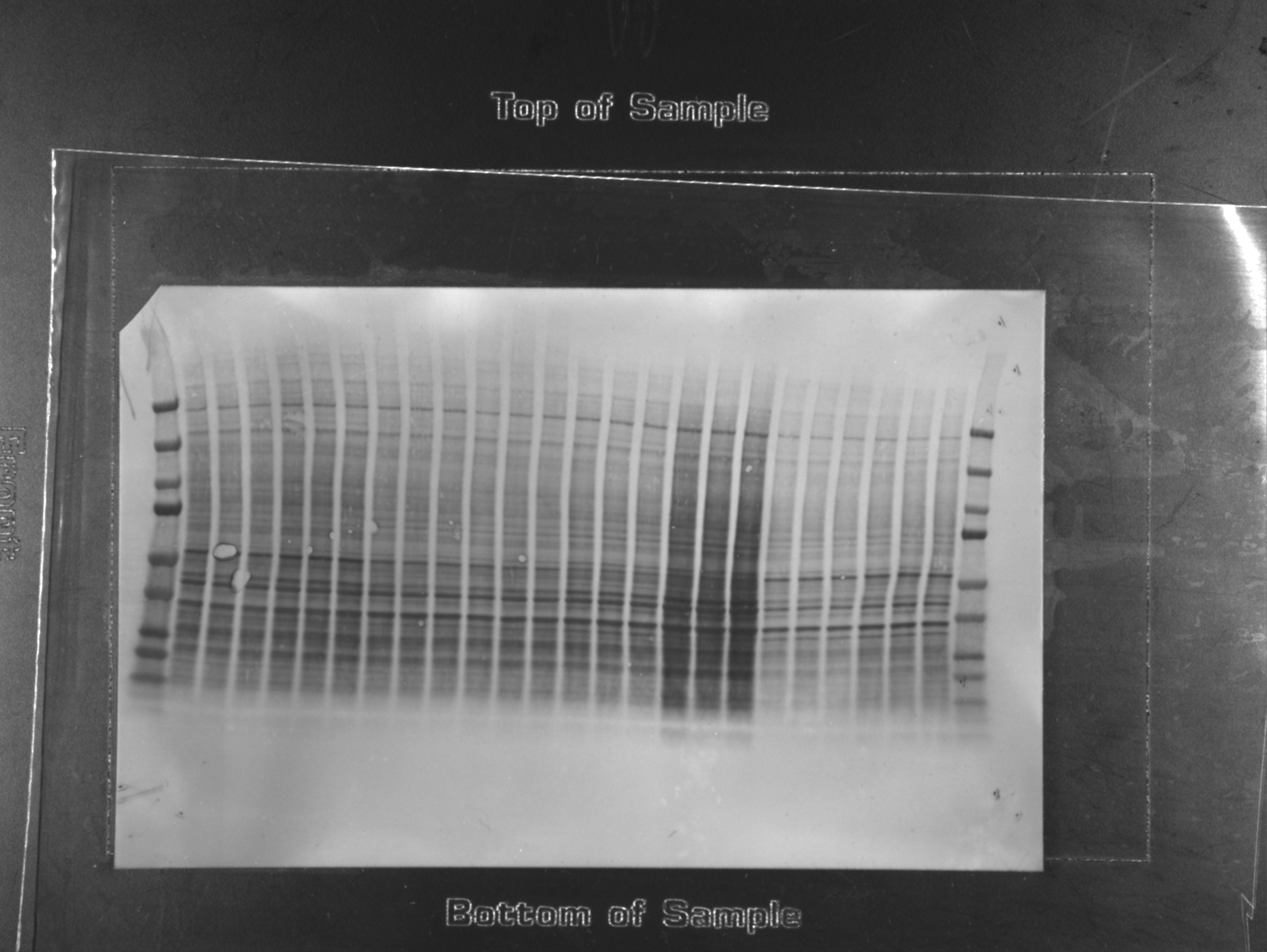

Supplement: Supplementary file 1 [file ijms-27-06233-s001.zip › Total protein ENOS LG HG COMBINED-As-Displayed.tif]

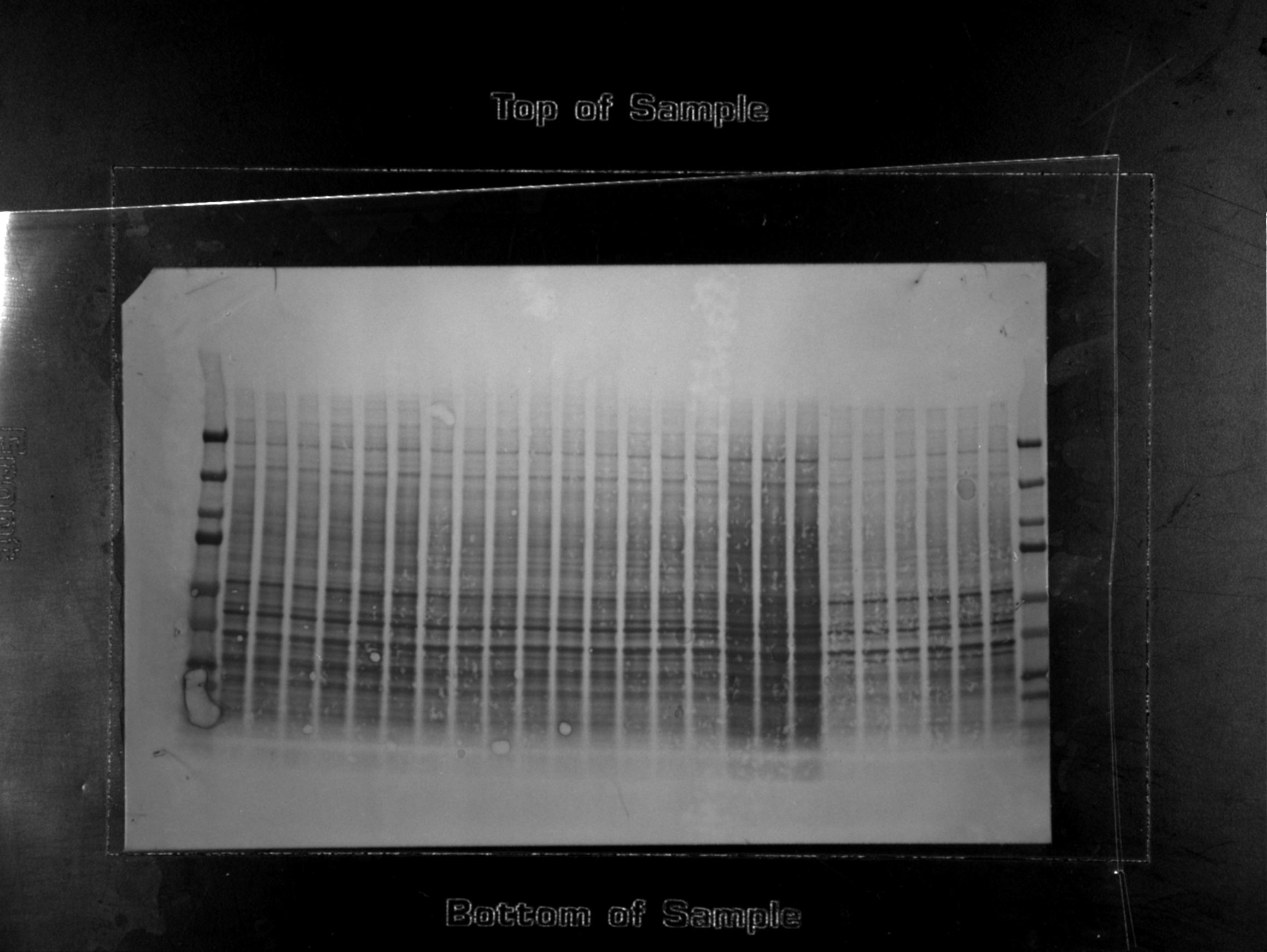

Supplement: Supplementary file 1 [file ijms-27-06233-s001.zip › Total protein VCAM1 CombinedLG HG membrane-As-Displayed(1).tif]

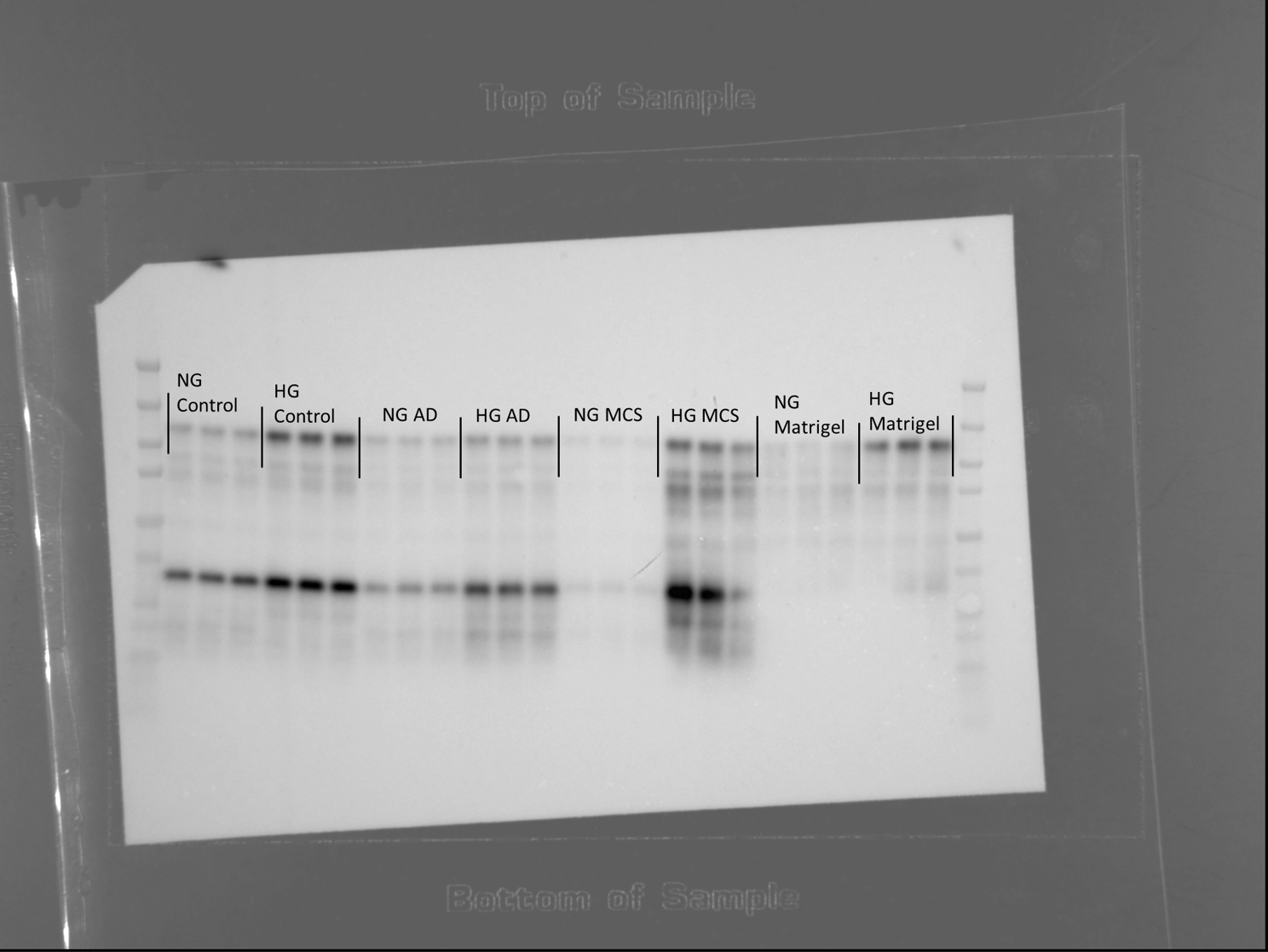

Supplement: Supplementary file 1 [file ijms-27-06233-s001.zip › CDH5 bands.png]

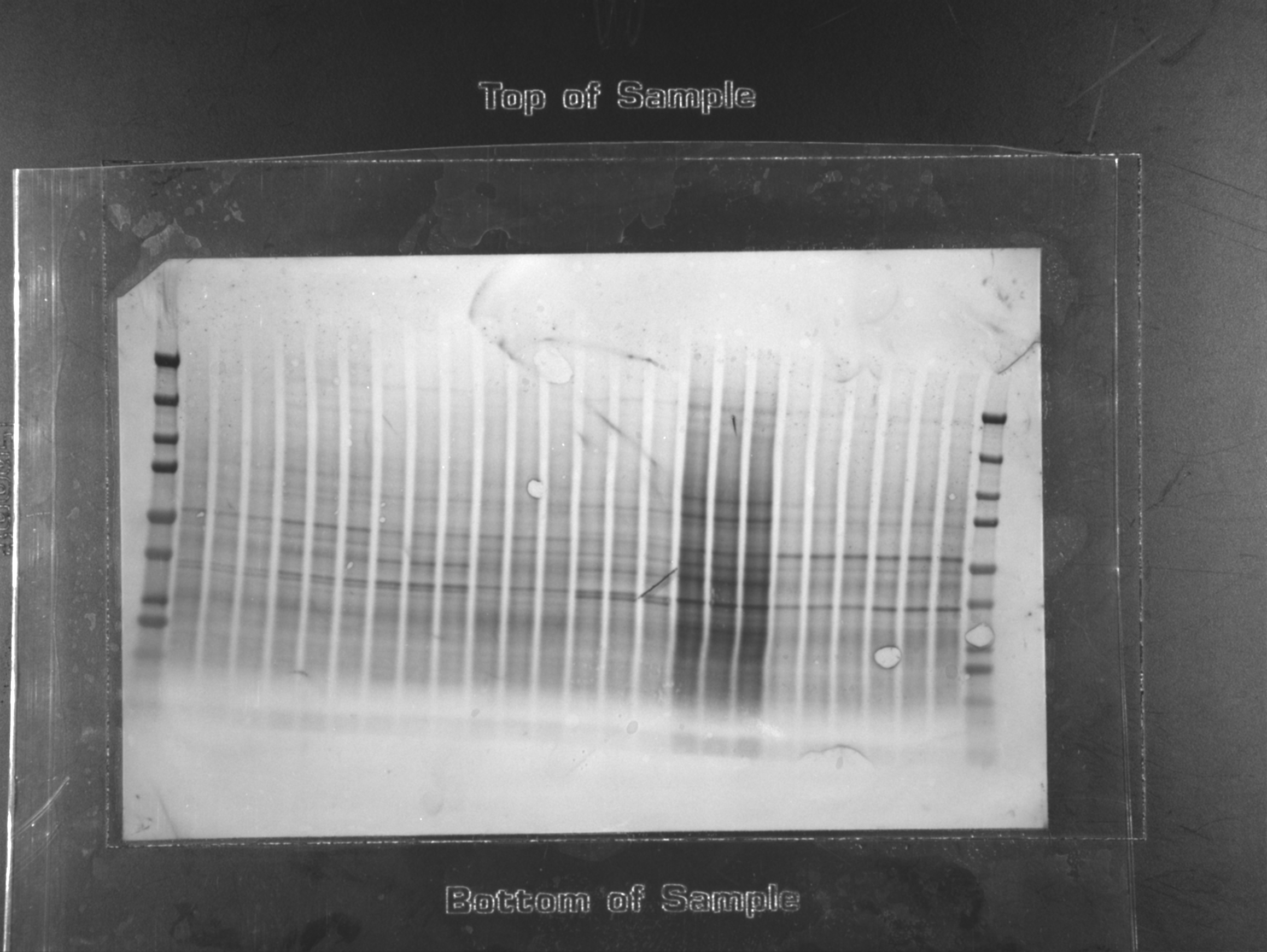

Supplement: Supplementary file 1 [file ijms-27-06233-s001.zip › 2026-06-03-102547-2026-06-03-102547-TP LG HG E cad-As-Displayed.tif]
